# Interleukin-33 Increases Antibacterial Defense by Activation of Inducible Nitric Oxide Synthase in Skin

**DOI:** 10.1371/journal.ppat.1003918

**Published:** 2014-02-20

**Authors:** Changwei Li, Hongquan Li, Ziwei Jiang, Tian Zhang, Yue Wang, Zhiheng Li, Yelin Wu, Shizhao Ji, Shichu Xiao, Bernhard Ryffel, Katherine A. Radek, Zhaofan Xia, Yuping Lai

**Affiliations:** 1 Shanghai Key Laboratory of Regulatory Biology, School of Life Sciences, East China Normal University, Shanghai, China; 2 Burn Institute of Chinese PLA and Department of Burn Surgery, Changhai Hospital, Second Military Medical University, Shanghai, China; 3 CNRS, UMR7355, University of Orleans, Orleans, France; 4 Department of Surgery, Burn and Shock Trauma Research Institute, Loyola University Chicago, Health Sciences Campus, Maywood, Illinois, United States of America; Columbia University, United States of America

## Abstract

Interleukin-33 (IL-33) is associated with multiple diseases, including asthma, rheumatoid arthritis, tissue injuries and infections. Although IL-33 has been indicated to be involved in *Staphylococcus aureus* (*S. aureus*) wound infection, little is known about how IL-33 is regulated as a mechanism to increase host defense against skin bacterial infections. To explore the underlying intricate mechanism we first evaluated the expression of IL-33 in skin from *S. aureus*-infected human patients. Compared to normal controls, IL-33 was abundantly increased in skin of *S. aureus*-infected patients. We next developed a *S. aureus* cutaneous infection mouse model and found that IL-33 was significantly increased in dermal macrophages of infected mouse skin. The expression of IL-33 by macrophages was induced by staphylococcal peptidoglycan (PGN) and lipoteichoic acid (LTA) via activation of toll-like receptor 2(TLR2) –mitogen-activated protein kinase (MAPK)-AKT-signal transducer and activator of transcription 3(STAT3) signaling pathway as PGN and LTA failed to induce IL-33 in *Tlr2*-deficient peritoneal macrophages, and MAPK,AKT, STAT3 inhibitors significantly decreased PGN- or LTA-induced IL-33. IL-33, in turn, acted on macrophages to induce microbicidal nitric oxygen (NO) release. This induction was dependent on inducible nitric oxide synthase (iNOS) activation, as treatment of macrophages with an inhibitor of iNOS, aminoguanidine, significantly decreased IL-33-induced NO release. Moreover, aminoguanidine significantly blocked the capacity of IL-33 to inhibit the growth of *S. aureus*, and IL-33 silencing in macrophages significantly increased the survival of *S. aureus* in macrophages. Furthermore, the administration of IL-33-neutralizing antibody into mouse skin decreased iNOS production but increased the survival of *S. aureus* in skin. These findings reveal that IL-33 can promote antimicrobial capacity of dermal macrophages, thus enhancing antimicrobial defense against skin bacterial infections.

## Author Summary

Interleukin-33 (IL-33) is associated with multiple diseases, including asthma, rheumatoid arthritis, tissue injuries and infections. Although IL-33 has been indicated to be involved in wound infections, little is known about how IL-33 is regulated as a mechanism to increase host defense against skin bacterial infections. Here we have shown that *Staphylococcus aureus* (*S. aureus*) cutaneous infection increases IL-33 expression in dermal macrophages in the skin. The expression of IL-33 by macrophages is induced by staphylococcal peptidoglycan (PGN) and lipoteichoic acid (LTA) via activation of toll-like receptor 2(TLR2) –mitogen-activated protein kinase (MAPK)-AKT-signal transducer and activator of transcription 3(STAT3) signaling pathway. IL-33 in turn acts on macrophages to inhibit the growth of *S. aureus* by binding to its receptor ST2 followed by activation of the AKT-β-catenin pathway, thus inducing and activating inducible nitric oxygen synthase (iNOS) to release microbiocidal nitric oxygen (NO). These findings reveal that IL-33 can promote antimicrobial capacity of dermal macrophages, thus enhancing antimicrobial defense against skin bacterial infections.

## Introduction

Interleukin-33 (IL-33), previously known as a nuclear factor from high endothelial venules [1], is a chromatin-associated nuclear cytokine from the IL-1 family that functions as an “alarmin” [2], [3]. It has been shown to be constitutively expressed in the nuclei of endothelial and epithelial cells *in vivo* [4] or to be released into the extracellular space after tissue damage [5], [6] to stimulate innate immune responses. Recently emerging evidence has shown that IL-33 is also a potent inducer of pro-inflammatory cytokines and chemokines by mast cells [7], [8], resulting in the development or exacerbation of asthma or atopic allergy and anaphylaxis [9]. In addition to functioning as an alarmin or inducer of proinflammatory cytokines, IL-33 plays an important protective role during parasitic infection via the induction of type 2 immune responses [10], [11] or promotes neutrophil proliferation and recruitment against *Staphylococcus aureus* (*S.aureus*) wound infection [12]. Although IL-33 has been shown to play a significant role during infection, little is known about how IL-33 is regulated as a mechanism to increase host defense against skin bacterial infections.

*S.aureus* skin infection is a major bacterial infection of the skin and has become an enormous public health problem due to the emergence of methicillin-resistant *S.aureus* (MRSA). Without treatment, *S.aureus* skin infection can disseminate and promote life-threatening infections. Remarkably, in the United States the estimated number of deaths caused by MRSA infection is around 18,500 per year, exceeding the number of deaths associated with human immunodeficiency virus infection/acquired immunodeficiency syndrome (HIV/AIDS) [13], [14]. Due to this rapidly emerging epidemic and the growing problem of antibiotic resistance, it is necessary to understand protective immune responses against *S.aureus* skin infection, thereby developing immune-based antibacterial therapies to combat *S.aureus* infection.

Recently several cytokines have been shown to enhance an effective immune response against *S.aureus* infection [14]. For example, IL-17 from skin γδ T cells stimulates keratinocytes to produce pro-inflammatory cytokines, chemokines and adhesion molecules that mediate neutrophil recruitment to the site of *S.aureus* infection to promote bacterial clearance [15]. IL-1 family cytokines IL-1α and IL-1β initiate an IL-1 receptor signaling loop to produce neutrophil-attracting chemokines, such as chemokine (C-X-C-motif) ligand 1 (CXCL1), CXCL2 and CXCL8 in human keratinocytes, leading to neutrophil recruitment to the site of *S.aureus* infection [16]. Furthermore, IL-18, another IL-1 family cytokine, protects burn-injured mice from MRSA by enhancing neutrophil function [17]. However, whether IL-33, a new member of IL-1 family, will regulate innate immune responses other than neutrophil functions against *S.aureus* skin infection remains largely unknown.

Given that the emergence of antibiotic-resistant *S.aureus* requires the development of new antibacterial therapies, and IL-1 family cytokines play important protective roles in *S.aureus* infections, we set out to investigate whether IL-33 is expressed after *S.aureus* cutaneous infection and determine if it exhibits a significant functional relevance in this system. Our findings uncover a vital protective role of IL-33 against *S.aureus* infection and delineate a previously unknown mechanism in host defense.

## Results

### *Staphylococcus aureus* infection induces IL-33 expression in skin

**Figure 1:**
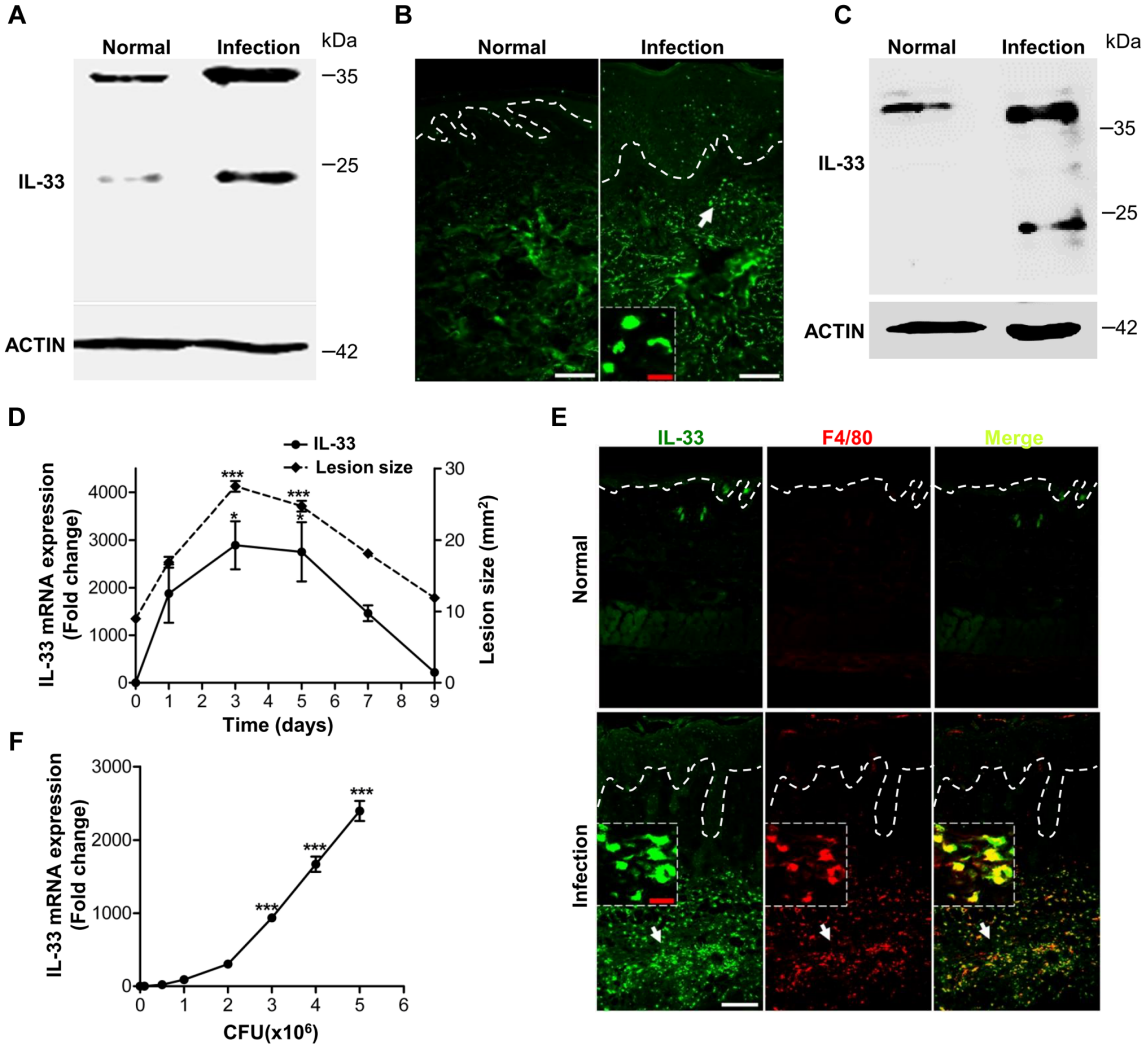
IL-33 expression in skin after *Staphylococcus aureus* infection. (**A**) Western blot of IL-33 in skin extracts from *S.aureus*-infected patients. Samples from 3 *S.aureus*-infected patients with abscess were pooled together. (**B**) Immunofluorescence analysis of IL-33 in skin of *S.aureus*-infected patients. This image is representative of three patients with abscess. Dotted white lines indicate the location of dermal-epidermal junction. Red scale bar represents 10 µm and white scale bars represent 50 µm. The arrow designates region of 200× magnification shown in inset. (**C**) Western blot of IL-33 in skin extracts from *S.aureus*-infected mice. Samples from 3 *S.aureus*-infected mice were pooled together. (**D**) Quantification of IL-33 expression in skin and the lesional size of skin lesions after mouse back skin infected with 1–2×10^7^ CFU *S.aureus*. Normal skin without infection was collected as control. (**E**) Immunofluorescence analysis of IL-33 and F4/80 (macrophage marker) in mouse skin after *S.aureus* infection. This image is representative of six mice with abscess. Dotted white lines indicate the location of dermal-epidermal junction. Red scale bar represents 10 µm and white scale bar represents 50 µm. Arrows designate region of 200× magnification shown in insets. (**F**) IL-33 expression in RAW264.7 cells stimulated with different doses of heat-inactivated *S.aureus* for 24 hours. *P<0.05 and ***P<0.001. *P* values were analyzed by one-way analysis of variance (ANOVA). Data are the means ± SEM and representative of two to three independent experiments with *n* = 3–6 per group.

Although IL-33 has been shown to be involved in host defense against intestinal nematode infection [11], [18] and *S.aureus* wound infection [12], the underlying intricate mechanism by which IL-33 is regulated to protect the host from bacterial skin infection remains largely unknown. To explore the role of IL-33 in bacterial infection we first evaluated the expression of IL-33 in skin from three *S.aureus*-infected human patients. Compared to normal controls, IL-33 was abundantly increased in skin of *S.aureus*-infected patients (Figure 1A). The immunofluorescence analysis showed that IL-33 protein was expressed both in epidermis and dermis of *S.aureus*-infected human skin (Figure 1B). To further confirm that the increase in cutaneous IL-33 production demonstrates a causal relationship with *S.aureus* infection, we evaluated IL-33 expression in a *S.aureus* cutaneous infection mouse model and found that both protein and mRNA of IL-33 were markedly increased in infected mouse skin (Figure 1C–1E). The formation of skin lesions caused by *S.aureus* was time-dependent and reached its peak at day-3 post-infection (Figure 1D). Consistent with skin lesions, the induction of IL-33 mRNA was time-dependent with the maximum induction observed in day-3-infected skin (Figure 1D). IL-33 protein expression was detectable by immunofluorescent staining primarily localized to dermal macrophages in infected skin (Figure 1E). Furthermore, to determine which cell type is a major producer of IL-33 during skin infection we used heat-inactivated *S.aureus* to stimulate primary keratinocytes, mast cells, neutrophils and macrophages *in vitro*. Heat-inactivated *S.aureus* significantly increased IL-33 mRNA in macrophages (Figure 1F) but not in mast cells and neutrophils (Figure S1A and S1B). To our surprise, in primary murine and human keratinocytes heat-inactivated *S.aureus* slightly induced IL-33 mRNA expression, but it markedly increased IL-33 protein production (Figure S1C–S1F). Taken together, these data demonstrate that *S.aureus* infection increases IL-33 in epidermal keratinocytes and dermal macrophages.

### PGN and LTA are two major molecules of *Staphylococcus aureus* to induce IL-33 in macrophages

**Figure 2:**
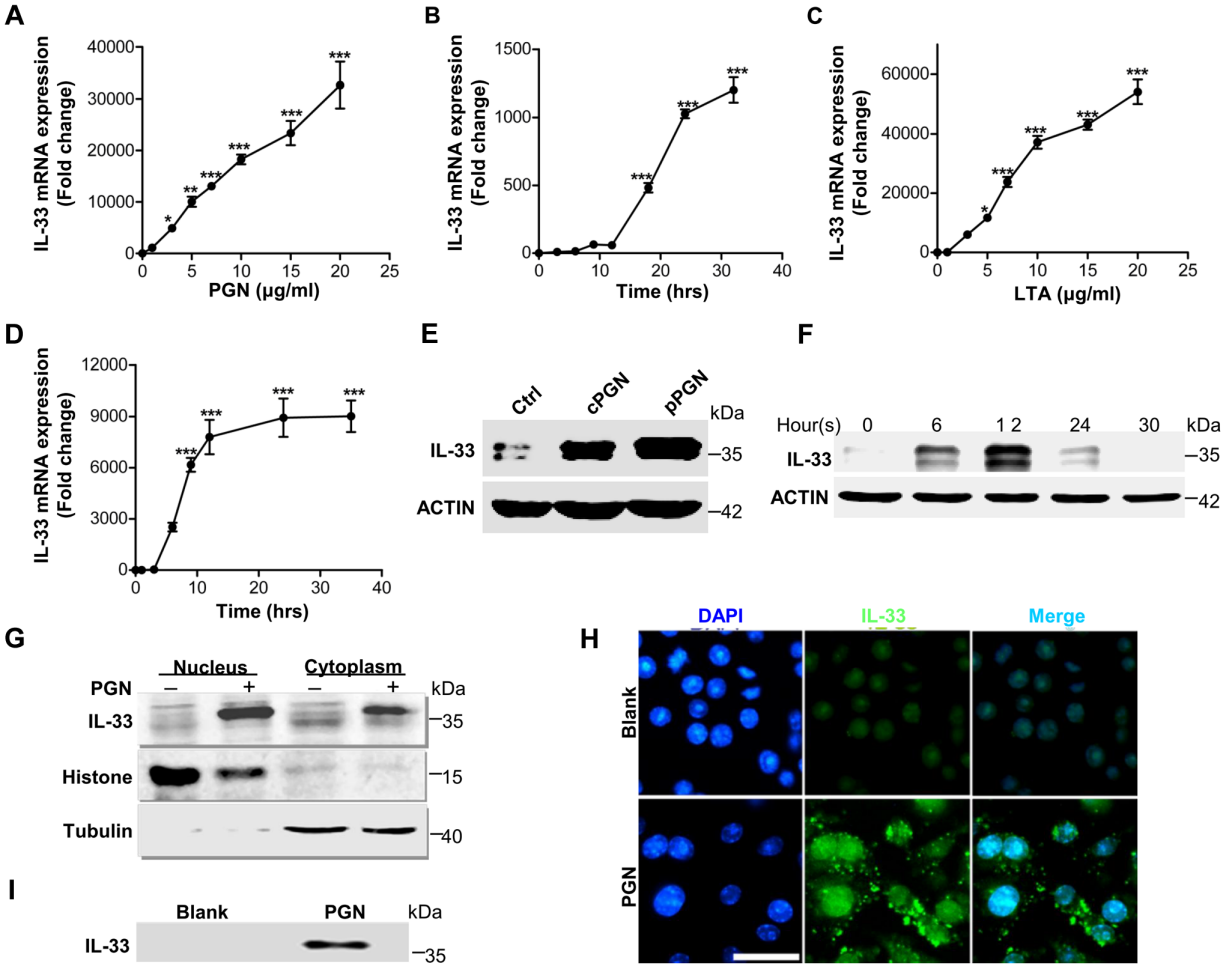
IL-33 expression induced by *S.aureus*-derived PGN and LTA in macrophages. (**A**&**B**) Quantification of IL-33 expression in macrophages RAW264.7 treated with different doses of PGN (**A**) or treated with 10 µg/ml PGN for different times (**B**). (**C**&**D**) Quantification of IL-33 expression in macrophages RAW264.7 treated with different doses of LTA (**C**) or treated with 10 µg/ml LTA for different times (**D**). (**E**) Western blot of IL-33 induced by commercial PGN (cPGN) and PGN purified from *S.aureus* CMCC(B)26003 (pPGN) in total cell lysate of primary peritoneal macrophages. (**F**) Western blot of IL-33 induced by PGN in total cell lysate of primary peritoneal macrophages at different time points. (**G**) Western blot of IL-33 from the cytoplasm or nucleus of macrophages RAW264.7 treated with 10 µg/ml PGN. Histone was used as an endogenous control for nuclear proteins and tubulin was used as an endogenous control for cytoplasmic proteins. (**H**) Immunofluorescent staining of IL-33 in macrophages RAW264.7 treated with PGN for 24 hours. Scale bar represents 50 µm. (**I**) IL-33 in cell culture media. Cell culture media of macrophages RAW264.7 treated with 10 µg/ml PGN was incubated with protein G beads coupled with IL-33 antibody for overnight. Next day, IL-33 captured by IL-33 antibody was eluted from beads for western blot. *P<0.05 and ***P<0.001. *P* values were determined by one-way ANOVA. Data are the means ± SEM of *n* = 3 and representative of three independent experiments.

We have observed that *S.aureus* infection enhanced IL-33 expression in keratinocytes and macrophages, next we sought to identify which molecules from *S. aureus* would be inducers of IL-33. Since *S.aureus* induced both mRNA and protein of IL-33 consistently in macrophages (Figure 1C–1F), and peptidoglycan (PGN) and lipoteichoic acid (LTA) are two major constituents of *S.aureus* cell wall, we thereby used *S.aureus*-derived PGN and LTA to stimulate macrophages *in vitro*. PGN, as well as LTA, significantly induced IL-33 mRNA in time- and dose- dependent manners (Figure 2A–2D). In addition to mRNA, both commercial PGN and PGN purified from *S.aureus* CMCC(B)26003, the strain used to infect mice, increased full-length IL-33 protein in pure primary peritoneal macrophages by western blot analysis (Figure 2E and 2F, Figure S2A). This production of IL-33 was detected in both the nucleus and cytoplasm following the nucleocytoplasmic separation analysis (Figure 2G). The immunofluorescence analysis also confirmed this observation that PGN induced the expression of IL-33 in both cytoplasm and nucleus (Figure 2H). Furthermore, IL-33 was detectable in cell culture media (Figure 2I). Taken together, these results show that PGN and LTA are two major molecules from *S.aureus* to directly induce IL-33 in macrophages.

### *Staphylococcus aureus* activates TLR2-MAPK-AKT-STAT3 signaling pathway to induce IL-33

**Figure 3:**
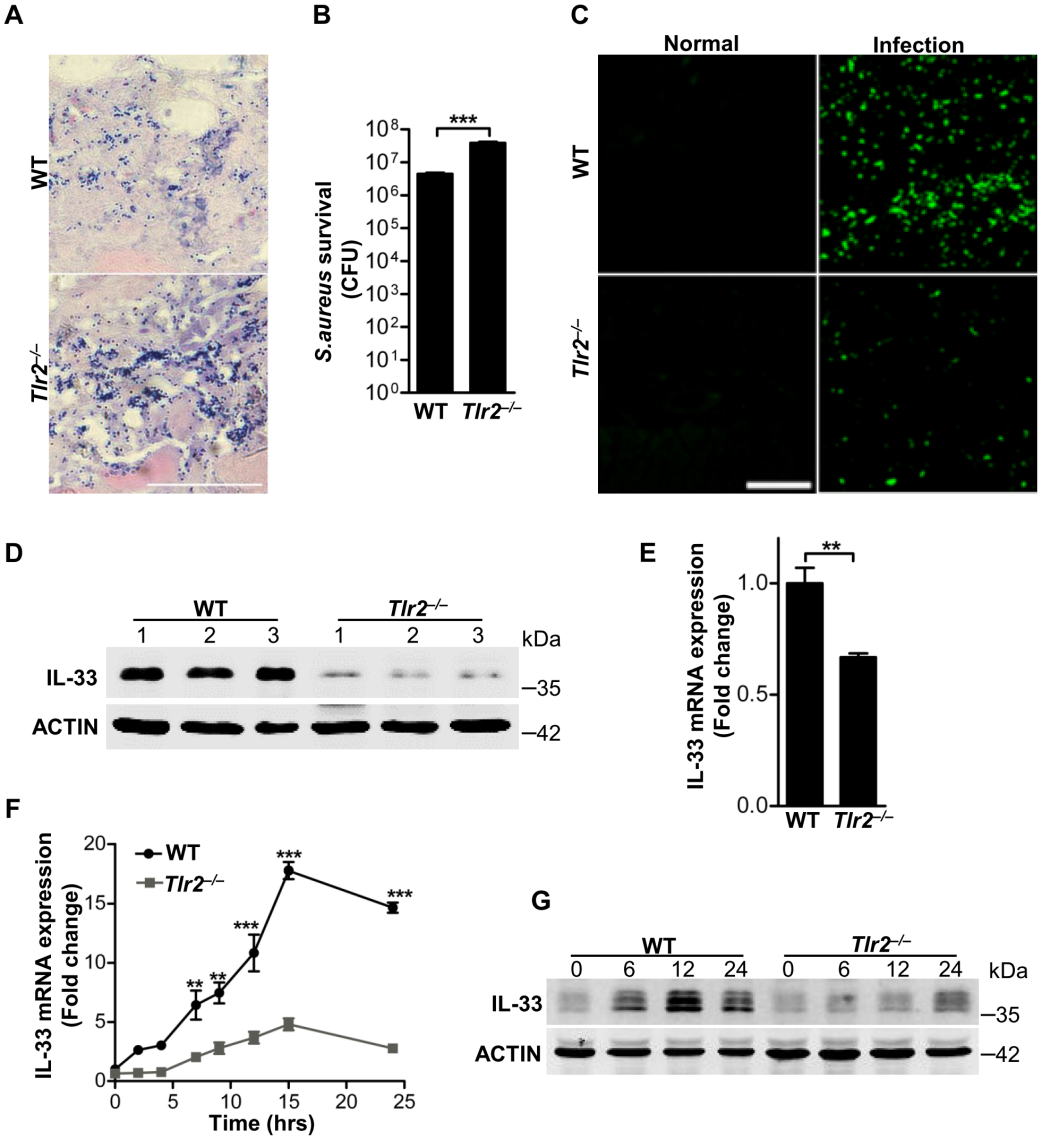
TLR2 is required for *Staphylococcus aureus* to induce IL-33. (**A**) Gram-positive staining of mouse skin infected with *S.aureus*. Scale bar represents 50 µm. (**B**) Local *S.aureus* survival in skin lesions at day-3 post-infection. (C) Immunofluorescent staining of IL-33 in wild-type and *Tlr2^−/−^* mouse skin infected with *S.aureus*. This image is representative of six mice with abscess. Scale bar represents 50 µm. (**D**) Western blot of IL-33 in skin lesions caused by *S.aureus* infection at 3 days. 1, 2, 3 represent three mice of each group were used. (**E**) Quantification of IL-33 mRNA expression in skin of wild-type and *Tlr2^−/−^* mice infected with *S.aureus*. (**F**) IL-33 mRNA expression in primary peritoneal macrophages from wild-type and *Tlr2^−/−^* mice stimulated with 10 µg/ml PGN. (**G**) Western blot of IL-33 in primary peritoneal macrophages from wild-type and *Tlr2^−/−^* mice stimulated with 10 µg/ml PGN for different times. **P<0.01 and ***P<0.001. *P* values were analyzed by two-tailed t tests in (**B** & **E**) or two-way ANOVA in (**F**). All data are representative of two independent experiments with *n* = 6 per group and are means ± SEM.

Having identified PGN and LTA from *S.aureus* as two major inducers of IL-33 in macrophages, we next sought to explore the molecular mechanisms involved in the induction of IL-33 by PGN and LTA in macrophages. Since Toll-like receptor 2 (TLR2) is a well-known receptor of Gram-positive bacteria, we hypothesized that the activation of TLR2 was required for *S.aureus* to induce IL-33. To test this, we infected wild-type (WT) and *Tlr2*-deficient (*Tlr2^−/−^*) mice with *S.aureus*. Compared to wild-type mice, the bacterial burden and survival of *S.aureus* was much higher in *Tlr2^−/−^* mice (Figure 3A and 3B), while both mRNA and protein of IL-33 were decreased in infected skin of *Tlr2^−/−^* mice (Figure 3C–3E). Furthermore, PGN induced IL-33 mRNA and protein in a time-dependent manner in isolated primary peritoneal macrophages from wild-type mice, but not in *Tlr2^−/−^* primary peritoneal macrophages (Figure 3F and 3G). In addition to PGN, LTA significantly induced IL-33 mRNA expression in wild-type but not *Tlr2^−/−^* primary peritoneal macrophages (Figure S2B). Altogether, these data demonstrate that *S.aureus* signals through TLR2 to induce IL-33 expression in macrophages.

**Figure 4:**
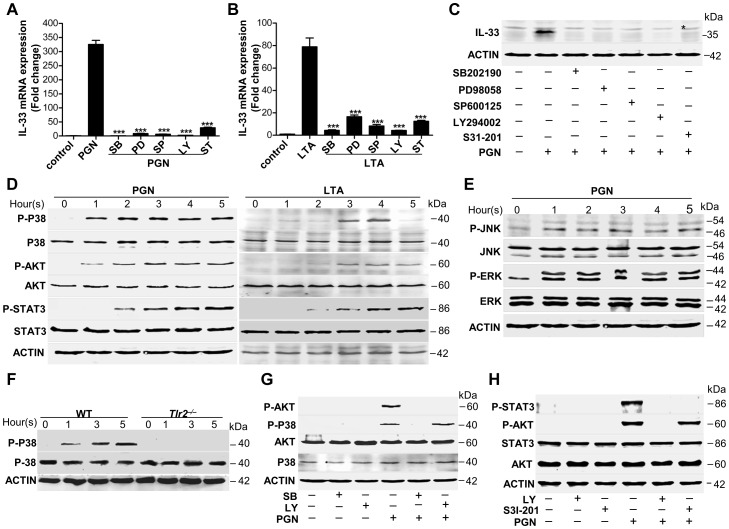
*Staphylococcus aureus* activates MAPKs-AKT-STAT3 signaling to induce IL-33. (**A**&**B**) Quantification of IL-33 mRNA of macrophages RAW264.7 treated with 10 µg/ml PGN (**A**) or LTA (**B**) in the presence or absence of different inhibitors. SB: p38 MAPK inhibitor SB202190 (5 µM); PD: MEK1 inhibitor PD98059 (20 µM); SP: JNK inhibitor SP600125 (15 µM); Ly: AKT inhibitor Ly294002 (50 µM); ST: STAT3 inhibitor S31-201 (50 µM). (**C**) Western blot of IL-33 in macrophages RAW264.7 stimulated by PGN in the presence or absence of inhibitors. * Unspecific bands. (**D**) Phosphorylation of p38 MAPK (Thr180/Tyr182), AKT (Ser473) and STAT3 (Tyr705) after RAW264.7 cells treated with 10 µg/ml PGN or LTA for 1–5 h. (**E**) Phosphorylation of JNK (Thr183/Tyr185) and ERK (Thr202/Tyr204) after RAW264.7 cells treated with 10 µg/ml PGN for 1–5 h. (**F**) Phosphorylation of p38 MAPK (Thr180/Tyr182) after wild-type and *Tlr2^−/−^* primary peritoneal macrophages treated with 10 µg/ml PGN for 1–5 h. (**G**) Phosphorylation of p38 MAPK (Thr180/Tyr182) and AKT (Ser473) after macrophages RAW264.7 treated with 10 µg/ml PGN in the presence of p38MAPK inhibitor SB202190 or AKT inhibitor Ly294002. (**H**) Phosphorylation of AKT (Ser473) and STAT3 (Tyr705) after macrophages RAW264.7 treated with 10 µg/ml PGN in the presence of AKT inhibitor Ly294002 or STAT3 inhibitor S31-201. ***P<0.001. *P* values were analyzed by one-way ANOVA in (**A**&**B**). All data are representative of two experiments with *n* = 3 per group and are means ± SEM.

Next we further explored TLR2-mediated downstream signaling pathways in macrophages. We used inhibitors of several key pathways downstream of TLR2 to treat macrophages in the presence or absence of PGN or LTA. Among these inhibitors, mitogen-activated protein kinase (MAPK) inhibitors, AKT inhibitor and signal transducers and activators of transcription 3 (STAT3) inhibitor significantly decreased the expression of PGN- or LTA-induced both mRNA and protein of IL-33 (Figure 4A–4C). PGN and LTA induced phosphorylation of p38 MAPK, AKT and STAT3 in a time-dependent manner in macrophages (Figure 4D). Besides p38 MAPK, PGN increased the phosphorylation of c-Jun N-terminal kinase (JNK) and extracellular signal-regulated kinase (ERK) (Figure 4E). Furthermore, PGN induced phosphorylation of p38 MAPK in wild-type peritoneal macrophages, but this effect was lost in *Tlr2^−/−^* peritoneal macrophages (Figure 4F); p38-MAPK inhibitor dampened PGN-induced AKT phosphorylation while AKT inhibitor did not have the capacity to block PGN-induced p38-MAPK phosphorylation (Figure 4G). Finally, AKT inhibitor prevented PGN-induced STAT3 phosphorylation, but not vice versa (Figure 4H). Taken together, these results suggest that *S.aureus* activates the TLR2-MAPK-AKT-STAT3 signaling pathway to regulate the production of IL-33 in macrophages.

### IL-33 activates ST2-AKT-β-catenin to induce iNOS

**Figure 5:**
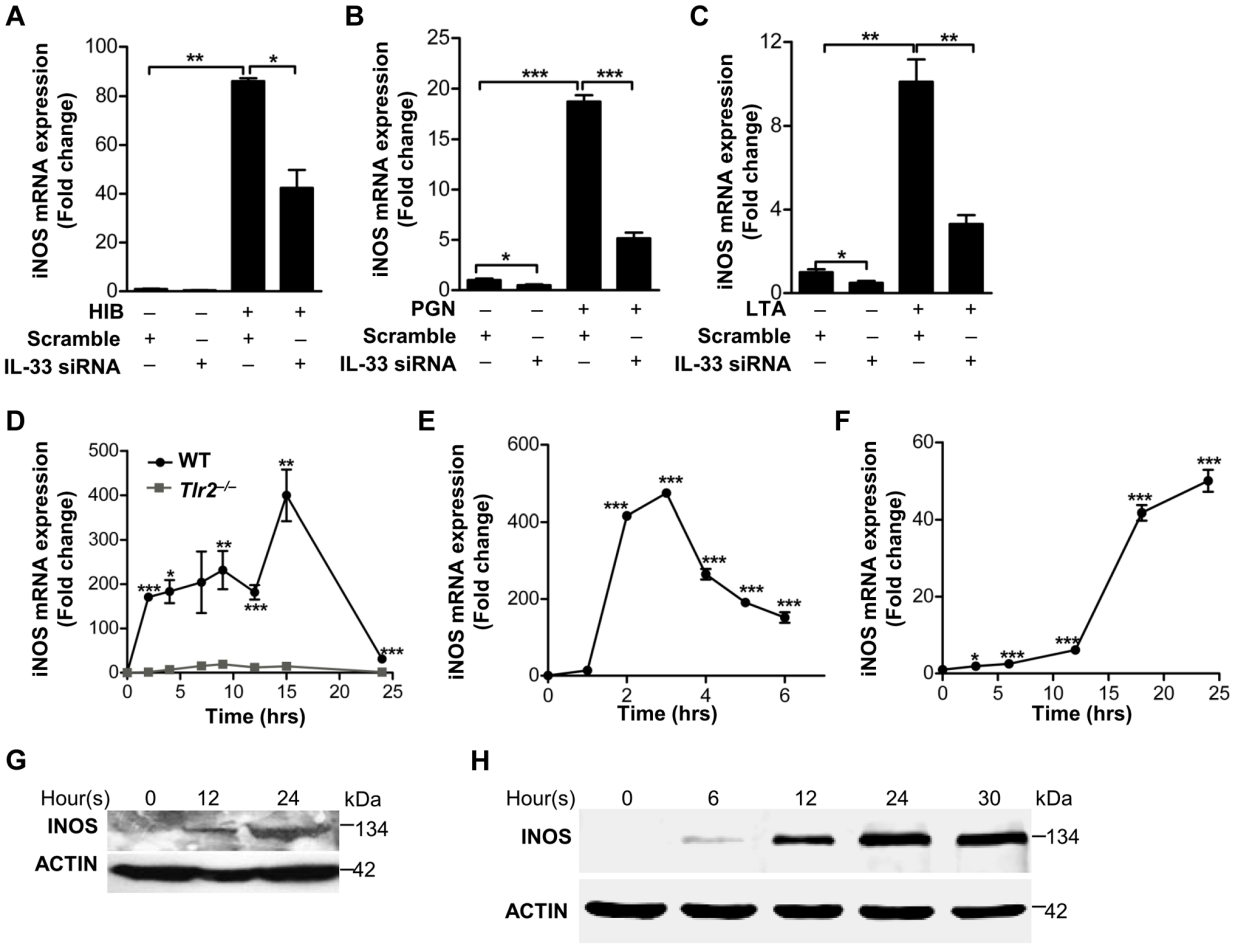
IL-33 induces iNOS in macrophages. (**A**–**C**) iNOS expression induced by heat-inactivated *S.aureus* (**A**) or PGN (**B**) or LTA(**C**) before or after IL-33 was silenced in primary peritoneal macrophages. (**D**) Quantification of iNOS mRNA expression of WT and *Tlr2^−/−^* primary peritoneal macrophages treated with 10 µg/ml PGN. (**E&F**) iNOS mRNA expression in BMDMs treated with 30 ng/ml full-length IL-33(**E**) or in primary peritoneal macrophages treated with 30 ng/ml processed IL-33(Ser109-Ile266) (**F**) for various times. (**G**&**H**) Western blot of iNOS in BMDMs treated with 30 ng/ml full-length IL-33(**G**) or in primary peritoneal macrophages treated with 30 ng/ml processed IL-33(Ser109-Ile266) (**H**) for various times. *P<0.05, **P<0.01 and ***P<0.001. *P* values were determined by one-way ANOVA (**A–C, E&F**) or two-way ANOVA (**D**). All data are means ± SEM of *n* = 3 and representative of two to three independent experiments.

Since we have delineated a key mechanism by which *S.aureus* induces IL-33 in macrophages, we next wanted to establish an *in vivo* role of IL-33 in *S.aureus* skin infection. Previous observations have suggested that IL-1 induces nitric oxygen synthase (NOS) to release nitric oxygen (NO), and IL-33 has been shown to induce NO production in endothelial cells [19], [20], [21]. Since we observed that heat-inactivated *S.aureus*, PGN and LTA stimulated the production of inducible form of NOS (iNOS) and NO in both time- and dose- dependent manners in macrophages (Figure S3), we hypothesized that IL-33 might be an intermediate for *S.aureus* to induce iNOS and NO in macrophages. To test this, we first determined if heat-inactivated *S.aureus* induced the expression of iNOS after IL-33 gene silencing. Heat-inactivated *S.aureus* significantly increased the expression of iNOS, and this increase was significantly inhibited after IL-33 was silenced using IL-33 siRNAs (Figure 5A). Consistent with this observation, iNOS induced by PGN and LTA was also significantly decreased after IL-33 was silenced (Figure 5B and 5C). Moreover, *S.aureus* completely failed to induce iNOS expression in *Tlr2^−/−^* primary peritoneal macrophages compared to that in wild-type primary peritoneal macrophages (Figure 5D), and the inactivation of MAPKs, AKT and STAT3 significantly decreased the expression of PGN- and LTA-induced iNOS (Figure S4A and S4B). To further confirm that IL-33 can directly active iNOS, we stimulated macrophages with recombinant full-length IL-33 and processed IL-33 (Ser109-Ile266) *in vitro* and found that both mRNA and protein of iNOS was induced by recombinant full-length and processed IL-33 in a time-dependent manner in primary peritoneal macrophages(Figure 5E–5H). Consistently, recombinant processed IL-33 increased both mRNA and protein of iNOS in time- and dose-dependent manners in macrophage cell line RAW264.7 (Figure S4C–S4E). Collectively, these data confirm that IL-33 is an important cytokine induced by *S.aureus* to increase iNOS expression.

**Figure 6:**
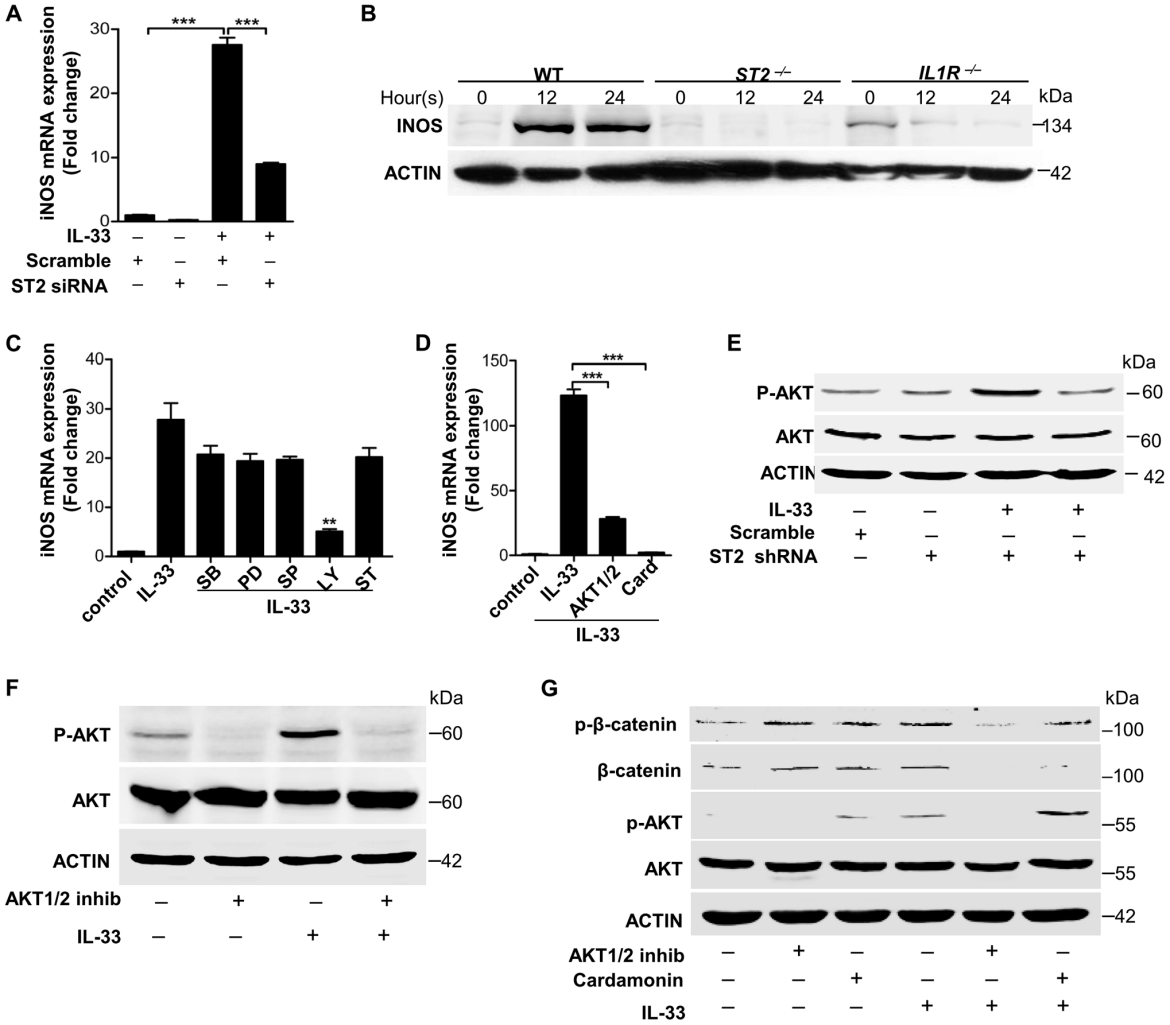
IL-33 activates ST2-AKT-β-catenin to induce iNOS. (**A**) iNOS mRNA expression induced by 30 ng/ml processed IL-33(Ser109-Ile266) before or after ST2 was silenced in primary peritoneal macrophages. (**B**) Western blot of iNOS in WT, *ST2^−/−^* and *IL1R^−/−^* primary peritoneal macrophages treated with 30 ng/ml processed IL-33(Ser109-Ile266) for different times. (**C**) iNOS mRNA expression induced by 30 ng/ml processed IL-33(Ser109-Ile266) in the presence or absence of different inhibitors. SB: p38 MAPK inhibitor SB202190 (5 µM); PD: MEK1 inhibitor PD98059 (20 µM); SP: JNK inhibitor SP600125 (15 µM); Ly: AKT inhibitor Ly294002 (50 µM); ST: STAT3 inhibitor S3I-201 (50 µM). (**D**) iNOS mRNA expression induced by 30 ng/ml processed IL-33(Ser109-Ile266) in the presence or absence of AKT1/2 specific inhibitor (8 µM) or β-catenin inhibitor cardamonin (9 µM). (**E**) Phosphorylation of AKT (Ser473) induced by 30 ng/ml processed IL-33(Ser109-Ile266) before and after ST2 was silenced in primary peritoneal macrophages. (**F**) Phosphorylation of AKT (Ser473) induced by 30 ng/ml processed IL-33(Ser109-Ile266) in the presence or absence of AKT1/2 specific inhibitor (8 µM) in primary peritoneal macrophages. (**G**) Phosphorylation of AKT (Ser473) and β-catenin (Ser675) induced by 30 ng/ml processed IL-33(Ser109-Ile266) in the presence or absence of AKT1/2 specific inhibitor (8 µM) or β-catenin inhibitor cardamonin (9 µM) in BMDMs. The phosphorylation of β-catenin at Ser675 resulted in the accumulation of total β-catenin in cells. **P<0.01 and *** P<0.001. *P* values were determined by one-way ANOVA. All data are means ± SEM of *n* = 3 and representative of two independent experiments.

It has been reported that IL-33 mediates its biological effects via its receptor ST2 [1]. We observed that recombinant processed IL-33 significantly increased ST2 in time- and dose-dependent manners in macrophages (Figure S5A and S5B). Silencing of ST2 significantly decreased the expression of iNOS induced by recombinant processed IL-33 (Figure 6A). Moreover, recombinant processed IL-33 induced iNOS protein production in WT peritoneal macrophages and bone marrow-derived macrophages (BMDMs), while this induction was completely or partially abrogated in *ST2^−/−^* or *IL1R^−/−^* peritoneal macrophages and BMDMs (Figure 6B and Figure S5C). To identify the key downstream pathways of ST2 activated by IL-33, we treated macrophages with multiple inhibitors in the presence or absence of recombinant processed IL-33. Among this inhibitors, AKT inhibitors (Ly294002 and the AKT1/2 specific inhibitor) and β-catenin inhibitor (cardamonin) significantly inhibited iNOS expression by recombinant processed IL-33 (Figure 6C and 6D). To confirm that AKT is a critical downstream signaling molecule for ST2, we next evaluated if recombinant processed IL-33 activated AKT after ST2 gene silencing. Recombinant processed IL-33 markedly increased the phosphorylation of AKT in a time-dependent manner (Figure S5D and S5E) and this increase was markedly inhibited after ST2 was silenced using ST2 shRNAs (Figure 6E and Figure S5F). Furthermore, AKT inhibitors completely inhibited IL-33-induced AKT phosphorylation or β-catenin phosphorylation and accumulation while β-catenin inhibitor failed to block IL-33-induced AKT phosphorylation (Figure 6F and 6G, Figure S5G). All these data demonstrate that IL-33 activates the ST2-AKT-β-catenin signaling pathway to regulate iNOS in macrophages.

### IL-33 activates iNOS to release NO against *Staphylococcus aureus* infection

**Figure 7:**
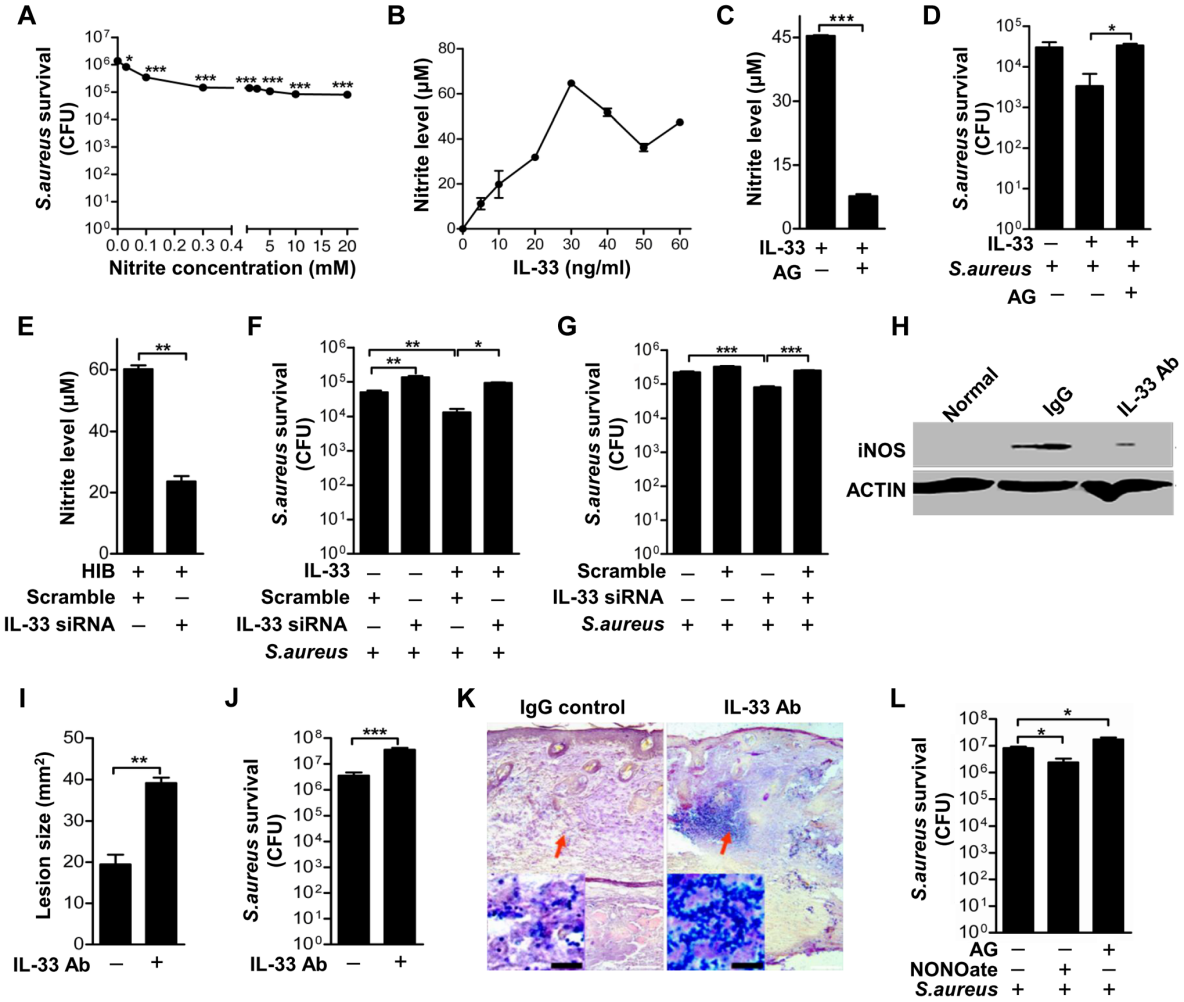
IL-33 activates iNOS to release NO against *Staphylococcus aureus* infection. (**A**) Acidified nitrite inhibited *S.aureus* growth *in vitro*. (**B**) Different doses of processed IL-33(Ser109-Ile266) induced NO release in RAW264.7 cells. (**C**) The release of NO by 30 ng/ml processed IL-33(Ser109-Ile266) in the presence or absence of iNOS inhibitor aminoguanidine. (**D**) The survival of *S.aureus* in the intracellular of RAW264.7 cells after stimulated with 30 ng/ml processed IL-33(Ser109-Ile266) in the presence or absence of aminoguanidine. (**E**) The release of NO by heat-inactivated *S.aureus* after IL-33 was silenced. (**F**) The survival of *S.aureus* in RAW264.7 cells stimulated by 30 ng/ml processed IL-33(Ser109-Ile266) before and after IL-33 was silenced. (**G**) The survival of *S.aureus* in RAW264.7 cells after treated with 30 ng/ml processed IL-33(Ser109-Ile266) in the presence or absence of AKT inhibitor Ly294002 (50 µM). (**H**) Western blot of iNOS in skin lesions caused by *S.aureus* infection at days 3 with or without IL-33 blockade. (**I**) Image J analysis of the lesion size of skin lesions treated as in (**H**). (**J**) Local *S.aureus* survival in skin lesions treated as in (**H**). (**K**) Gram-positive staining of *S.aureus* in infected skin of (**H**). (**L**) Local *S.aureus* survival in skin of mice treated with NO-donor NONOate or iNOS inhibitor aminoguanidine. *P<0.05, **P<0.01 and ***P<0.001. *P* values were determined by one-way ANOVA in (**A**, **D**, **F**, **G** and **L**) or two-tailed t tests in (**C**, **E**, **I** and **J**). All data are representative of two to three independent experiments with *n* = 3–6 per group and are means ± SEM.

To understand the immunological relevance of iNOS induced by IL-33 and infection, we next set out to elucidate the biological role of IL-33. It is known that iNOS regulates NO production and NO plays an important role in host defense against intracellular pathogens [22], [23]. Furthermore, it has been reported that topically application of NO is a potentially useful preventive and therapeutic strategy against superficial skin infections, including MRSA skin infection [24]. Consistent with these observations, we observed that acidified nitrite significantly inhibited *S.aureus* growth *in vitro* (Figure 7A). To test whether the activation of iNOS by IL-33 would release NO against *S.aureus* infection, we first investigated the capacity of IL-33 to induce NO. Recombinant processed IL-33 induced NO release in a dose-dependent manner (Figure 7B). This induction was dependent on iNOS activation, as treatment of macrophages with a relatively selective inhibitor of iNOS, aminoguanidine, significantly decreased NO release (Figure 7C). Moreover, aminoguanidine significantly blocked the capacity of IL-33 to inhibit the growth of intracellular *S.aureus* in macrophages (Figure 7D), and IL-33 silencing in macrophages not only significantly decreased the production of NO induced by *S.aureus* (Figure 7E) but also increased the survival of *S.aureus* in macrophages (Figure 7F). Since we have shown that the induction of IL-33 by *S.aureus* (including PGN and LTA) or iNOS by IL-33 was dependent on AKT activation, we next used the AKT inhibitor to block the production of IL-33 or NO. We found that the AKT inhibitor significantly increased the survival of *S.aureus* and blocked the capacity of IL-33 to inhibit the growth of *S.aureus* (Figure 7G).

To further address the role of IL-33 in *S.aureus* infection, we treated mice with IL-33 neutralizing antibody before mice were intradermally injected with *S.aureus*. *S.aureus* infection increased iNOS production in macrophages (Figure 7H, Figure S6A) and neutrophil recruitment (Figure S6B) while the blockage of IL-33 by its neutralizing antibody markedly inhibited macrophage-derived iNOS production (Figure 7H and Figure S6A) and neutrophil recruitment (Figure S6B). Consistent with this, mice treated with IL-33 neutralizing antibody exhibited larger infectious skin lesions when compared with control mice injected with IgG control (Figure 7I). Mouse skin treated with IL-33 neutralizing antibody exhibited greater *S.aureus* survival at the local site of infection (Figure 7J) and a greater *S.aureus* bacterial burden in skin lesion (Figure 7K). Moreover, the presence of an iNOS inhibitor increased *S.aureus* survival in mouse skin, while treatment with NO-donor DETA-NONOate reduced the bacterial load *in vivo* (Figure 7L). Thus, these data confirm that IL-33 activates iNOS to release NO against *S.aureus* infection.

## Discussion

Innate immunity is the first line of defense to protect the host from bacterial skin infection. IL-33, as a cytokine from innate immune cells, has been shown to be protective against parasitic and bacterial infection [10], [11], [12], but the underlying intricate mechanism by which IL-33 is regulated to increase host defenses against bacterial skin infection remains largely unknown. Here we observed that IL-33 increased host innate defense via the induction and activation of iNOS to release microbicidal NO. Our results demonstrate that PGN and LTA, two major cell wall components of *S.aureus*, induce IL-33 via activation of TLR2-MAPK-AKT-STAT3 signaling pathway. Furthermore, the intradermal administration of IL-33 neutralizing antibody or iNOS inhibitor in skin decreases NO release, which leads to increased survival of *S.aureus*, while the direct administration of NO-donor DETA-NONOate decreases the bacterial survival in skin lesions. The mechanism for IL-33-mediated antibacterial function involves NO release through the binding to its receptor ST2, followed by the activation of AKT-β-catenin and iNOS, events we show have a major role in inhibiting *S.aureus* infection in skin. Therefore, the identification of IL-33 as a stimulus for NO, and the elucidation of its mechanism of induction and action, provides a new mechanism for understanding innate immune responses during bacterial skin infections. These findings also offer potential immune-based antibacterial therapies to combat bacterial skin infections by targeting IL-33 and downstream NO induction.

When a pathogen breaches the skin barrier, neutrophils are quickly recruited to the site of infection followed by the infiltration of macrophages. At the initial stage of the host immune response to infection, macrophages and neutrophils are generally thought to function primarily as phagocytic cells [25]. Although these two professional phagocytes possess overlapping and complementary characteristics during infection, their distinct antimicrobial properties that stem from their innate immune lineage make them irreplaceable as individual components of the innate immune system [26]. Neutrophils are equipped with an array of microbicidal mechanisms and employ several granular antimicrobial molecules, including defensins and cathelicidin to combat pathogenic infections [27], [28]. Macrophages exhibit remarkable phagocytic abilities compared to neutrophils, which enhance their contribution to direct antimicrobial activities [29]. Here we provide a previously unidentified mechanism by which macrophages exert their primary antimicrobial defense against *S. aureus* infection by induction of IL-33 to activate iNOS for NO release into the intracellular and extracellular milieu. In response to *S.aureus* infection, TLR2 present on the cell surface of macrophages allows for the recognition of PGN and LTA, followed by activation of p38 MAPK-AKT-STAT3 signaling to induce IL-33 production. In turn, IL-33 induces and activates iNOS to release NO in macrophages. This previously unknown function of IL-33 in macrophages sheds new light on the function of macrophages in innate host defense. Interestingly, the increase of IL-33 mRNA by PGN or LTA is more than 1000 fold higher in macrophage RAW264.7 while only 4–15 fold higher in primary peritoneal macrophages compared to vehicle treated, which might be due to the differences between cell line and primary cells. Besides induction of microbicidal NO, IL-33 recruits neutrophils to the site of *S.aureus* infection (Figure S6B) but does not induce iNOS in neutrophils (data not shown), which is consistent with the previous observation [12]. In addition to murine macrophages, we analyzed IL-33 expression induced by *S.aureus* in other cell types, including mast cells, neutrophils and keratinocytes. *S.aureus* markedly induces IL-33 protein production but slightly induces mRNA expression in keratinocytes (Figure S1C–S1F) or completely fails to induce IL-33 expression in neutrophils and mast cells. Therefore, our whole study is primarily focused on murine macrophages.

The expression of IL-33 is primarily localized to nonhematopoietic cells, particularly in endothelial and epithelial cells [2]. Full-length, unprocessed IL-33 has been shown to localize within the nucleus of epithelial and endothelial cells [4]. Here we show that IL-33 is also expressed in hematopoietic-derived macrophages as well as epidermal keratinocytes after *S.aureus* infection, although there is certain difference between mRNA and protein of IL-33 in epidermal keratinocytes (Figure S1C–S1F). It is known that in principle, unprocessed proteins of IL-1 family such as IL-1α should remain in the cellular compartment in which they are synthesized [30]. However, we observe that, upon PGN stimulation, full-length IL-33 is detectable not only inside macrophages (including cytoplasm and nucleus) but also in cell culture media. How full-length, unprocessed IL-33 is excreted from macrophages remains unclear. We speculate that full-length, unprocessed IL-33 is passively released by necrotic cells into the extracellular milieu as an alarmin molecule to indicate cellular stressors (i.e. infection or injury). Alternatively, *S.aureus* infection may induce acute extracellular accumulation of ATP, and this autocrine ATP activates purinergic receptors to induce full-length, unprocessed IL-33 release [31]. Moreover, both full-length IL-33 and processed IL-33 are detected in skin during *S.aureus* infection, while heat-inactivated *S.aureus* only induces full-length IL-33 in macrophages. One may assume that upon stimulation, neutrophils release cathepsin G and elastase to process full-length IL-33 to mature bioactive forms *in vivo* [5], [32] while *in vitro* macrophages fail to perform this process due to lack of the above proteases. However, it is also possible that full-length IL-33 is processed by an unknown mechanism *in vivo* that does not exist in macrophages. Furthermore, although the previous report shows that the activity of processed IL-33 in the induction of inflammatory cytokines is around 10-fold higher than full-length IL-33, in our system full-length IL-33 exhibits the similar activity as processed IL-33 in the induction of iNOS (Figure 5E–5H). In spite of this discrepancy, both data suggest that the functional domain of IL-33 is primarily at the C-terminus of IL-33.

Like other members of IL-1 family, IL-33 acts as a double-edged sword. Accumulating evidence shows that IL-33 has an important role during the development or exacerbation of airway inflammation. The direct administration of IL-33 to mouse lung induces IL-5-producing T cells, thus exacerbating allergen-induced airway inflammation [33]. In addition to the initiation of inflammation, IL-33 can effectively attenuate sepsis by mobilizing the innate cells, neutrophils, to the site of infection, helping to clear the pathogens [34]. The above evidence clearly shows that IL-33 has a dual role in different inflammatory disease processes. It is logical that IL-33 would exert these distinct functions depending on the immune mechanism underlying the pathogenesis of each disease condition. However, it is unknown whether this dual role of IL-33 exists in other skin diseases, such as atopic dermatitis. It has been shown that IL-33 mounts potent Th2 inflammatory responses and induces the degranulation of IgE-sensitized mast cells in skin, resulting in exacerbation of atopic dermatitis [35]. Usually, patients with atopic dermatitis exhibit greater colonization by *S.aureus*. Here, we show that *S.aureus* infection increases IL-33 expression in macrophages, and IL-33 in turn induces and activates iNOS to release NO, thus limiting bacterial growth and/or survival during skin infection. The different functions of IL-33 in mast cells and macrophages suggest that IL-33 may play a dual role during *S.aureus* infection in atopic dermatitis patients. Therefore, targeting IL-33 as an alternative treatment strategy for atopic dermatitis patients should be approached with caution. The key will be to evoke a reduction in the detrimental aspects of IL-33 without increasing the risk of infection in atopic dermatitis patients.

Nitric oxide, as a free radical gas molecule, exhibits potent antimicrobial activities against multiple bacteria including *S.aureus* [36], [37]. The administration of NO-donor compounds on *S.aureus*-infected soft tissue significantly inhibited the growth of *S.aureus* and effectively cleared infection in chronic wounds [36]. It is known that all major cell types in the skin, such as keratinocytes, fibroblasts, endothelial cells and macrophages, can produce NO under different stimuli [38], [39], [40]. Multiple cytokines, including IL-1β and IFNγ, have been shown to regulate iNOS activation to induce NO production; however, the administration of iNOS inhibitors exacerbates the overwhelming majority of infections [41], [42]. Here we observe that IL-33, a new member of the IL-1 family, stimulates NO production in macrophages via ST2-AKT-β-catenin-dependent iNOS activation, which is consistent with previous observations of endothelia NOS (eNOS) activation in endothelial cells [21], [43]. To our surprise, we have also observed that the induction of iNOS by IL-33 is dependent on IL-1R as well as ST2 in macrophages (Figure 6B and Figure S5C). One may assume that the deficiency of IL-1R impairs the function of its accessory protein (IL-1RAP), that forms a heterodimeric receptor complex with ST2 for IL-33 binding [9]. The other speculation is that IL-33 induces IL-1α and IL-1β (data not shown), and then IL-1α and IL-1β in turn activate IL-1R to induce iNOS in macrophages [20]. Furthermore, the release of NO by IL-33 is 10–60 µM (Figure 7B) while 30 µM NO *in vitro* inhibited around 50% of *S.aureus* growth (Figure 7A), suggesting that skin-derived NO induced by IL-33 might be sufficient to protect host against *S.aureus* infection. Moreover, treatment of macrophages with IL-33 leads to an increase in iNOS expression as well as NO production (Figure 5E–5H, and Figure 7B), while blockade of IL-33 by IL-33 neutralizing antibody or inhibition of iNOS with a specific inhibitor *in vivo* significantly prevents IL-33-induced bacterial clearance(Figure 7J–7L). Thus, the evidence presented here clearly shows that macrophage-derived NO induced by IL-33 plays a critical role in host innate defense against *S.aureus* infection. However, whether NO induced by IL-33 is biologically relevant or IL-33 activates other innate immune effectors to control *S.aureus* infection needs further investigation.

In conclusion, our study provides evidence that IL-33 is important to protect the host from bacterial skin infections, specifically *S.aureus* infection. IL-33 is an intermediate molecule embedded within the cross-talk between microbes and the host, and may function as a previously unknown key element in the treatment of *S.aureus* skin infection. Our findings implicate the potential of IL-33 as a therapeutic target in skin bacterial infections. The identification of IL-33 function in the skin provides new insights into pathways contributing to antimicrobial defense and may ultimately lead to the development of novel treatment regimens for skin infection and inflammation.

## Materials and Methods

### Human samples, mouse and bacterial strains

All human skin samples were obtained from 3 methicillin resistant *S.aureus*-infected adult patients (including one female and two male patients) in Changhai Hospital, Second Military Medical University, China. MRSA caused abscess in all these patients. 2 mm skin around lesional sites was taken for analyzing IL-33 expression and skin far from lesional sites was used as control. Due to the limited skin samples, protein from 3 patients was pooled together for western blot analysis. *Tlr2^−/−^* mouse breeding pair was purchased from the Jackson Laboratory (USA). C57BL/6 mice and age-matched *Tlr2^−/−^* mice were housed in the animal facility at East China Normal University. *ST2^−/−^* and *IL1R^−/−^* mice were housed in the animal facility at University of Orleans, France. *Staphylococcus aureus* CMCC(B)26003 with methicillin resistance was purchased from the National Center for Medical Culture Collections in China.

### Ethics statement

All human sample acquisitions were approved by the ethical committee of Changhai Hospital, Second Military Medical University, China, and performed in accordance with the declaration of Helsinki Principles. All participants provided written informed consent which was obtained before enrolment in the study.

All animal experiments were performed according to the protocol approved by the East China Normal University (ECNU) Animal Care and Use Committee and in direct accordance with Ministry of Science and Technology of the People's Republic of China on Animal Care guidelines. The protocol was approved by ECNU Animal Care and Use Committee (Protocol ID: AR2012/12017). All surgeries were performed under anesthesia and all efforts were made to minimize suffering.

### Cell culture and stimulation

RAW264.7 cells and mast cells (Chinese Academy of Sciences) were cultured in DMEM (Invitrogen) medium containing 10% FBS (GIBCO), 50 U.ml^−1^ penicillin and 50 ug.ml^−1^ streptomycin (GIBCO) under standard culture conditions. Neonatal human epidermal keratinocytes (Cascade biologics) were cultured as previously reported [44] and primary murine epidermal keratinocytes were isolated and cultured as our previous report [45]. Primary peritoneal neutrophils were isolated from six WT mice by mice by i.p. injection of 3 ml thioglycolate medium (Sigma). Cells were harvested 4 hours later by peritoneal lavage with cold PBS, followed by washing with RPMI1640 medium (GIBCO). Primary peritoneal macrophages were isolated from six to nine WT and *Tlr2^−/−^* mice by i.p. injection of 1 ml thioglycolate medium (Sigma). Cells were harvested 3 days later by peritoneal lavage with cold PBS, followed by washing with DMEM medium (GIBCO). After cultured in medium DMEM for 7 days, we did FACS analysis by using F4/80 antibody to determine the purity of primary peritoneal macrophages. Bone marrow-derived macrophages from 3 WT, *ST2^−/−^* and *IL1R^−/−^* mice were cultured as the standard protocol from Cold Spring Harbor Laboratory [46].

For all cell stimulation experiments, 2×10^5^ cells were seeded in each well of 24-well or 8×10^5^ cells were seeded in each well of 6-well plates. When cells were grown to 80% confluence, the indicated doses of heat-inactivated *S.aureus*, PGN (Sigma), LTA (InvivoGen), recombinant murine IL-33 (R&D) or human IL-33 (Sino Biological Inc) or different inhibitors under concentrations without cytotoxicity or low cytotoxicity (Figure S7A–S7D) were used to stimulate cells. After 24 hours treatment, cells were collected for RNA isolation or western blot.

### shRNA preparation and targeting gene knockdown

Oligonucleotides encoding mouse IL-33 siRNA and ST2 shRNA were designed. Blast search was performed by using the National Center for Biotechnology Information (NCBI) database to ensure that siRNAs or shRNA constructs were targeting only mouse IL-33 and ST2, respectively. For ST2 shRNA constructs, the oligonucleotides were annealed and cloned into the pLL3.7 vector according to the manufacturer. 4 ug of pLL3.7 constructs containing shRNAs, 4 ug of packaging plasmid psPAX2 (Addgene), and 2 ug of envelope plasmid pMD2.G (Addgene) were used to transfected HEK293T cells by calcium phosphate precipitation method. 48 h later, lentiviruses containing targeted gene shRNA were collected and used to transfect macrophages. Or IL-33 siRNAs were directly transfected with macrophages by using GenEscort transfection reagent (Wisegen Biotechnology Corporation). The cytotoxicity of siRNA and shRNA in macrophages was tested by MTT assay (Figure S7E–S7H)

### Real-time quantitative RT-PCR

Real-time RT-PCR specific primers were used to evaluate gene expression. RNA analysis was done as previously reported [45].

### Fractionation of cytoplasmic and nuclear proteins

Cells were treated with IL-33 for indicated times and then lysed by a buffer (pH = 7.4) containing 10 mM HEPES, 10 mM NaCl, 1 mM KH_2_PO_4_, 5 mM NaCO_3_, 5 mM EDTA-2Na, 1 mM CaCl_2_, 0.5 mM MgCl_2_, 1 mM PMSF, 1 mM NaF and NaVO_3_. The lysate was collected and added with 2 M sucrose, then centrifuged at 6500×g for 15 minutes (4°C). The supernatant containing the cytoplasmic fraction was harvested and the pellet containing the nuclear fraction was resuspended in a buffer (pH = 7.4) containing 10 mM Tris, 300 mM sucrose, 1 mM EDTA, 0.1% NP-40. The protein concentration was determined by BCA Protein Assay Kit (Thermo) for immunoblotting.

### Immunoblotting and immunofluorescent staining

2 mm mouse skin taken from the margin of murine wounds or cells stimulated as described was lysed by using RIPA buffer (pH 7.4) containing protease inhibitor cocktail (Roche). To concentrate IL-33 from cell culture medium, mouse IL-33 antibody (R&D) immunocomplexed to Protein A/G agarose (Abmart) was used to capture IL-33. 10 µg of total protein was used for Western blot. IL-33, iNOS, p38 MAPK, AKT and β-catenin were detected by immunoblot with IL-33 antibody (R&D), iNOS antibody (Abcam), p38 MAPK antibody (Cell Signaling), AKT antibody (Cell Signaling) and β-catenin antibody (Cell signaling), respectively.

5 µm of formalin-fixed, paraffin-embedded tissue sections was mounted on glass slides and used for immunohistochemistry and staining. 4% PFA was used to fixate the samples. After 10-min fixation and subsequent pretreated with antigen retrieval solution, the sections were stained with IL-33 (R&D) antibody or iNOS (Abcam) antibody and F4/80 antibody (Santa Cruz) or Ly6G/Gr antibody (Abcam). The sections were reprobed with anti-Goat IgG FITC (Jackson immunoresearch) or anti-Rat IgG TRITC conjugate antibody (KPL), and then mounted in ProLong Gold antifade reagent with DAPI (Invitrogen) and visualized them by the microscope (Leica).

### Determination of the microbiocidal activity of acidified nitrite

The microbiocidal activity of acidified nitrite was determined as previous reported [47]. Briefly, potassium nitrite solutions were prepared with final concentrations of 0, 0.03, 0.1, 0.3, 1, 2.5, 5, 10, 20 µmol ml^−1^. *S.aureus* was adjusted to 1×10^6^ CFU per microwell. The pH was adjusted to 4.5 by hydrochloric acidification. The microwells were filled in sequence with: 60 µl nitrite solution, 60 µl bacterial suspension and 120 µl acidified broth. Plates were covered with self-adhesive sterile Sealplates and incubated at 37°C for 5 hours. Bacteria from the microwells were plated on tryptic soy broth agar plates. The microbiocidal activity of acidified nitrite was determined by counting the bacterial colonies.

### iNOS inhibition

Macrophages were pretreated with 0.36 mM aminoguanidine (AG) (Sigma) for 3 hours before PGN, LTA or IL-33 stimulation. The nitrite concentration in the culture supernatant was assayed by NO assay kit (Nanjing Jiancheng biotechnology Ltd. Co) according to the manufacturer's instructions.

### In vitro killing assay

RAW264.7 cells were pretreated with 0.36 mM aminoguanidine (Sigma) for 3 hours before 30 ng ml^−1^ mIL-33 was added to stimulate cells for 20 hours. 10^6^ CFU *S.aureus* was added and co-incubated with cells for another 5 hours at 37°C. Bacteria from cell culture medium and lysate were plated on tryptic soy broth agar. The next day, bacterial colonies were counted.

### IL-33 neutralization in vivo

50 µg of monoclonal mouse IL-33 antibody (MBL) was intraperitoneally injected into each mouse 24 hours before infection. Next day, 50 µg of monoclonal mouse IL-33 antibody (MBL) was intradermally injected into mouse back skin. 4 hours later, 1–2×10^7^ CFU *S.aureus* was interadermally injected into second IL-33-injected sites. 3 days later, skin around the lesions was taken for CFU assay.

### Cutaneous infection in vivo

The backs of age-matched adults were shaved and hair was removed by using chemical depilation as previous described [45]. In the same day *S.aureus* was grown in Trypocase Soy Broth (TSB) for overnight. Next day, 1 mL of overnight culture of *S.aureus* was re-inoculated into 30 mL fresh TSB. When the bacterium grew to logarithmic phase (OD600 = 0.7–0.8), 50 µl of live *S.aureus* (1–2×10^7^ CFU) was complexed to the same volume of cytodex beads (Sigma) as carrier and then intradermally injected into mouse back skin where the iNOS inhibitor aminoguanidine (100 mg per kg body weight) or the NO donor DETA- NONOate (10 mg per kg body weight) was intradermally injected one hour before. Mice were euthanized at the indicated timepoints. In some experiments, skin around the lesions was collected and homogenized in 1× PBS to determine the number of surviving *S.aureus*. In other experiments, RNA or protein from normal or infected skin was collected either for real-time RT-PCR or for western blot, or stored in 4% paraformaldehyde for immunostaining (Shanghai Sanjing Gongmao Ltd.Co).

### Histopathological diagnose and detection of *Staphylococcus aureus*

Paraffin embedded skin sections were used by haematoxylin-eosin staining for the histopathological diagnosis, and modified Gram-staining [48] was used to detect *S.aureus* in infected lesions. This analysis was performed using conventional optical microscopy (Leica). The bacteria were characterized by coccoid characteristics.

### Statistical analysis

All data are present as mean ± SEM. We used two-tailed t tests to determine significances between two groups. For multiple groups, we employed a one-way or two-way ANOVA with Bonferroni post test using GraphPad prism version 5. For all statistical tests, we considered *P* value<0.05 to be statistically significant.

## Supporting Information

Figure S1 ***Staphylococcus aureus*** **induces the expression of IL-33 in different cell types in skin.** (**A**) The expression of IL-33 in neutrophils. 10^6^ CFU heat-inactivated *S.aureus* was used to stimulate neutrophils. 24 hours later, cells were collected for RNA isolation. (**B**) The expression of IL-33 in mast cells treated as in (**A**). (**C&D**) The quantification of IL-33 in primary human keratinocytes (**C**) or in primary murine keratinocytes (**D**) treated as in (**A**). (**E&F**) Western blot of IL-33 induced by 10^6^ CFU heat-inactivated *S.aureus* in primary murine keratinocytes (**E**) or primary human keratinocytes (**F**). **P<0.01, ***P<0.001. *P* values were analyzed by one-way ANOVA. Data are the means ± SEM and representative of two independent experiments with *n* = 3 per group.
(TIF)

Figure S2 ***Staphylococcus aureus*** **induces IL-33 in primary peritoneal macrophages.** (**A**) FACS analysis of the purity of primary peritoneal macrophages. Isolated primary peritoneal macrophages were stained with F4/80 antibody and then anti-rat Alexa Fluor 488 antibody (BioLegend). Afterwards, the cells were washed with DPBS and then resuspended at 1×10^6^/ml. Cell samples were then analyzed and sorted using a Calibut Flow cell sorter (BD biosciences) and FlowJo software (BD biosciences) for data acquisition and analysis. The purity of primary peritoneal macrophages is 94.7%. (**B**) IL-33 mRNA expression in primary peritoneal macrophages from wild-type and *Tlr2^−/−^* mice stimulated with 10 µg/ml LTA for different times. *P<0.05, ***P<0.001. *P* values were analyzed by two-way ANOVA. Data are the means ± SEM and representative of two independent experiments with *n* = 3 per group.
(TIF)

Figure S3 ***Staphylococcus aureus*** **induces iNOS and NO release.** (**A**) Western blot of iNOS in primary peritoneal macrophages stimulated by 10 µg/ml commercial PGN (cPGN) and PGN purified form *S.aureus* CMCC(B)26003 (cPGN) for 24 hours. (**B**) iNOS mRNA expression in RAW264.7 cells treated with different doses of heat-inactivated *S.aureus* for 24 hours. (**C**) NO production induced by different doses of heat-inactivated *S.aureus* for 24 hours in RAW264.7 cells. (**D&E**) Quantification of iNOS mRNA in RAW264.7 cells treated with different doses of PGN (**D**) or LTA (**E**) for 24 hours. (**F**) iNOS mRNA expression in RAW264.7 cells treated with 10 µg/ml PGN or LTA for various times. (**G**) NO production by different doses of PGN or LTA for 24 hours in RAW264.7 cells. ***P<0.001. *P* values were determined by one-way ANOVA. All data are means ± SEM of *n* = 3 and representative of two independent experiments.
(TIF)

Figure S4 **PGN, LTA and processed IL-33 induces iNOS expression in macrophages.** (**A&B**) iNOS mRNA expression induced by 10 µg/ml PGN (**A**) or LTA (**B**) in the presence or absence of different inhibitors in RAW264.7 cells. SB: p38 MAPK inhibitor SB202190 (5 µM); PD: MEK1 inhibitor PD98059 (20 µM); SP: JNK inhibitor SP600125 (15 µM); Ly: AKT inhibitor Ly294002 (50 µM); ST: STAT3 inhibitor S3I-201 (50 µM). (**C&D**) iNOS mRNA expression induced by 30 ng/ml process IL-33 for different times (**C**) or by different dose of process IL-33 for 24 hours (**D**) in RAW264.7 cells. (**E**) Western blot of iNOS induced by 30 ng/ml process IL-33 for different times in RAW264.7 cells. * *P*<0.05, **P<0.01 and ***P<0.001. *P* values were determined by one-way ANOVA. All data are means ± SEM of *n* = 3 and representative of two independent experiments.
(TIF)

Figure S5 **IL-33 induces iNOS via the activation of ST2-AKT-β-catenin in macrophages.** (**A&B**) Quantification of ST2 mRNA expression in macrophages treated with 30 ng/ml IL-33 for different times (**A**) or different doses of IL-33 for 24 hours (**B**). (**C**) Western blot of iNOS in WT, *ST2^−/−^* and *IL1R^−/−^* BMDMs treated with 30 ng/ml processed IL-33(Ser109-Ile266) for different times. (**D&E**) Western blot of AKT phosphorylation induced by 30 ng/ml processed IL-33 for different times in RAW264.7 cells (**D**) or primary peritoneal macrophages (**E**). (**F**) AKT phosphorylation induced by 30 ng/ml processed IL-33 before and after ST2 was knocked down in RAW264.7 cells. (**G**) AKT phosphorylation induced by 30 ng/ml processed IL-33 in the presence or absence of AKT inhibitor Ly294002 (50 µM) in RAW264.7. **P<0.01 and ***P<0.001. *P* values were determined by one-way ANOVA. All data are means ± SEM of *n* = 3 and representative of two independent experiments.
(TIF)

Figure S6 **IL-33 neutralization decreases iNOS in macrophages and the recruitment of neutrophils in skin lesions.** (**A**) Immunofluorescence analysis of iNOS and F4/80 (macrophage marker) in *S.aureus*-infected skin before and after IL-33 was neutralized. Red scale bar represents 10 µm and white scale bars represent 100 µm. The arrow designates region of 200× magnification shown in inset. (**B**) Immunofluorescence analysis of Ly6G/GR1 (neutrophil marker) in *S.aureus*-infected skin before and after IL-33 was neutralized. Scale bar represents 100 µm.
(TIF)

Figure S7 **The cytotoxicity of inhibitors and shRNAs.** (**A**) Cell viability of PGN, LTA or different inhibitors in macrophages RAW264.7 by MTT analysis. PGN: 10 µg/ml; LTA: 10 µg/ml; SB: p38 MAPK inhibitor SB202190 (5 µM); PD: MEK1 inhibitor PD98059 (20 µM); SP: JNK inhibitor SP600125 (15 µM); Ly: AKT inhibitor Ly294002 (50 µM); ST: STAT3 inhibitor S31-201 (50 µM). (**B**) Inhibitors failed to induce IL-33 protein in RAW264.7 cells by western blot analysis. (**C&D**) Cell viability of RAW264.7 cells treated with IL-33 in the presence or absence of different inhibitors. IL-33: 30 ng/ml; SB: p38 MAPK inhibitor SB202190 (5 µM); PD: MEK1 inhibitor PD98059 (20 µM); SP: JNK inhibitor SP600125 (15 µM); Ly: AKT inhibitor Ly294002 (50 µM); ST: STAT3 inhibitor S31-201 (50 µM); AKT1/2 specific inhibitor (8 µM); Card: β-catenin inhibitor cardamonin (9 µM). (**E–H**) Cell viability of RAW264.7 cells treated with 10^6^ CFU heat-inactivated *S.aureus* (**E**) or PGN(**F**) or LTA (**G**) before and after IL-33 was knocked down or treated with IL-33 before and after ST2 was knocked down (**H**). n.s. no significance. *P* values were determined by one-way ANOVA. All data are means ± SEM of *n* = 3 and representative of two independent experiments.
(TIF)
