# Supplementary figures and images for: Interleukin-33 Increases Antibacterial Defense by Activation of Inducible Nitric Oxide Synthase in Skin
Source: PLoS Pathog. 2014 Feb 20;10(2):e1003918. doi: 10.1371/journal.ppat.1003918 (PMC3930573; doi:10.1371/journal.ppat.1003918)

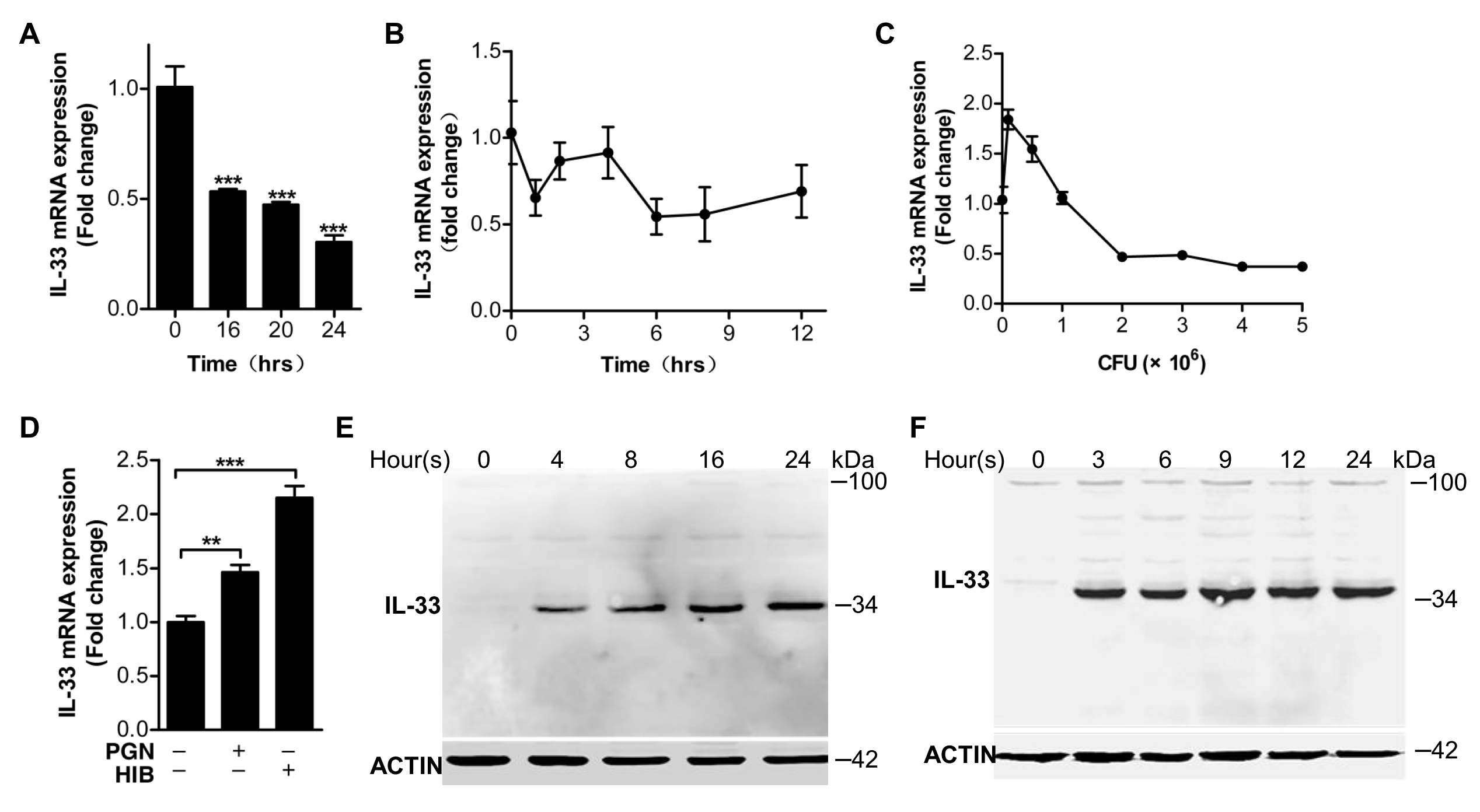

Supplement: Figure S1 — Staphylococcus aureus induces the expression of IL-33 in different cell types in skin. (A) The expression of IL-33 in neutrophils. 106 CFU heat-inactivated S.aureus was used to stimulate neutrophils. 24 hours later, cells were collected for RNA isolation. (B) The expression of IL-33 in mast cells treated as in (A). (C&D) The quantification of IL-33 in primary human keratinocytes (C) or in primary murine keratinocytes (D) treated as in (A). (E&F) Western blot of IL-33 induced by 106 CFU heat-inactivated S.aureus in primary murine keratinocytes (E) or primary human keratinocytes (F). **P<0.01, ***P<0.001. P values were analyzed by one-way ANOVA. Data are the means ± SEM and representative of two independent experiments with n = 3 per group. (TIF) [file ppat.1003918.s001.tif]

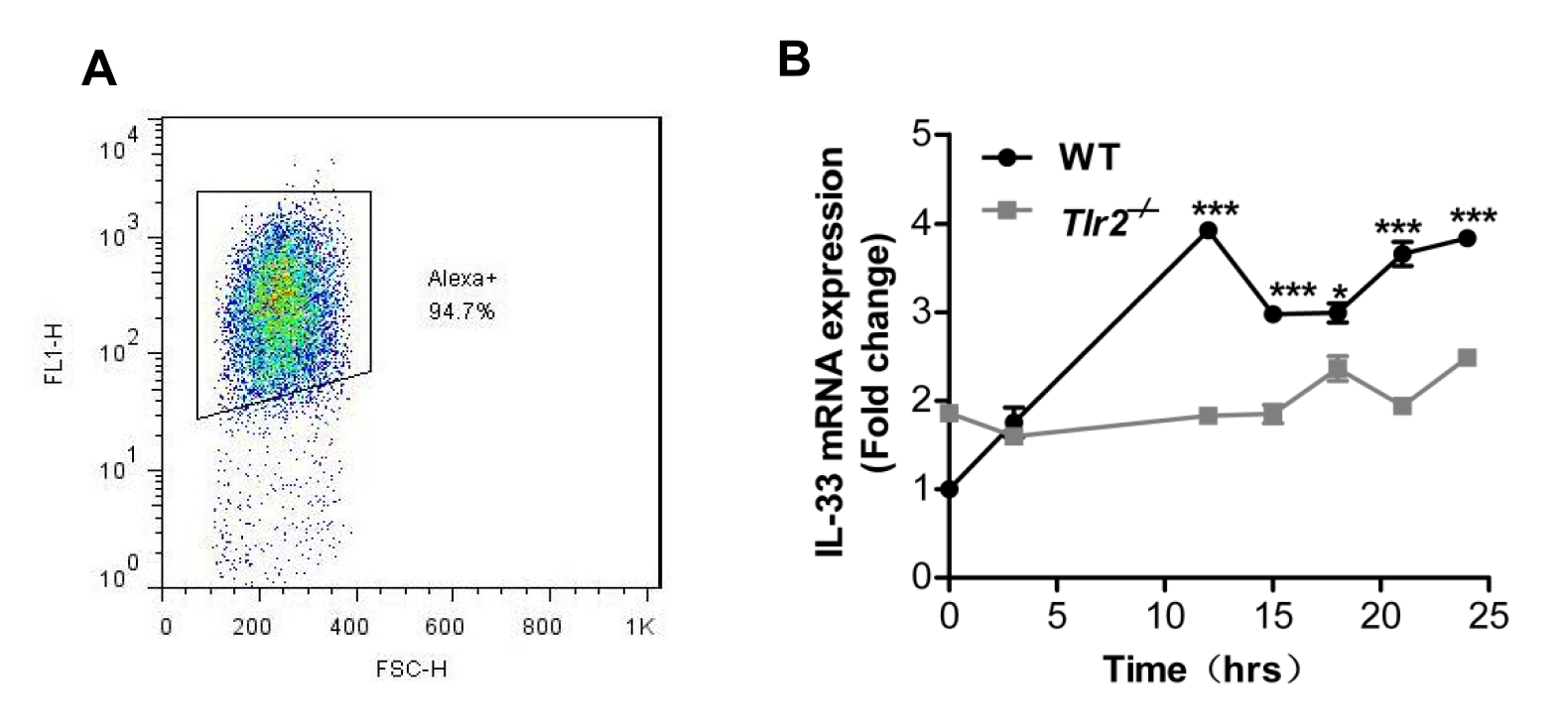

Supplement: Figure S2 — Staphylococcus aureus induces IL-33 in primary peritoneal macrophages. (A) FACS analysis of the purity of primary peritoneal macrophages. Isolated primary peritoneal macrophages were stained with F4/80 antibody and then anti-rat Alexa Fluor 488 antibody (BioLegend). Afterwards, the cells were washed with DPBS and then resuspended at 1×10^6^/ml. Cell samples were then analyzed and sorted using a Calibut Flow cell sorter (BD biosciences) and FlowJo software (BD biosciences) for data acquisition and analysis. The purity of primary peritoneal macrophages is 94.7%. (B) IL-33 mRNA expression in primary peritoneal macrophages from wild-type and Tlr2−/− mice stimulated with 10 µg/ml LTA for different times. *P<0.05, ***P<0.001. P values were analyzed by two-way ANOVA. Data are the means ± SEM and representative of two independent experiments with n = 3 per group. (TIF) [file ppat.1003918.s002.tif]

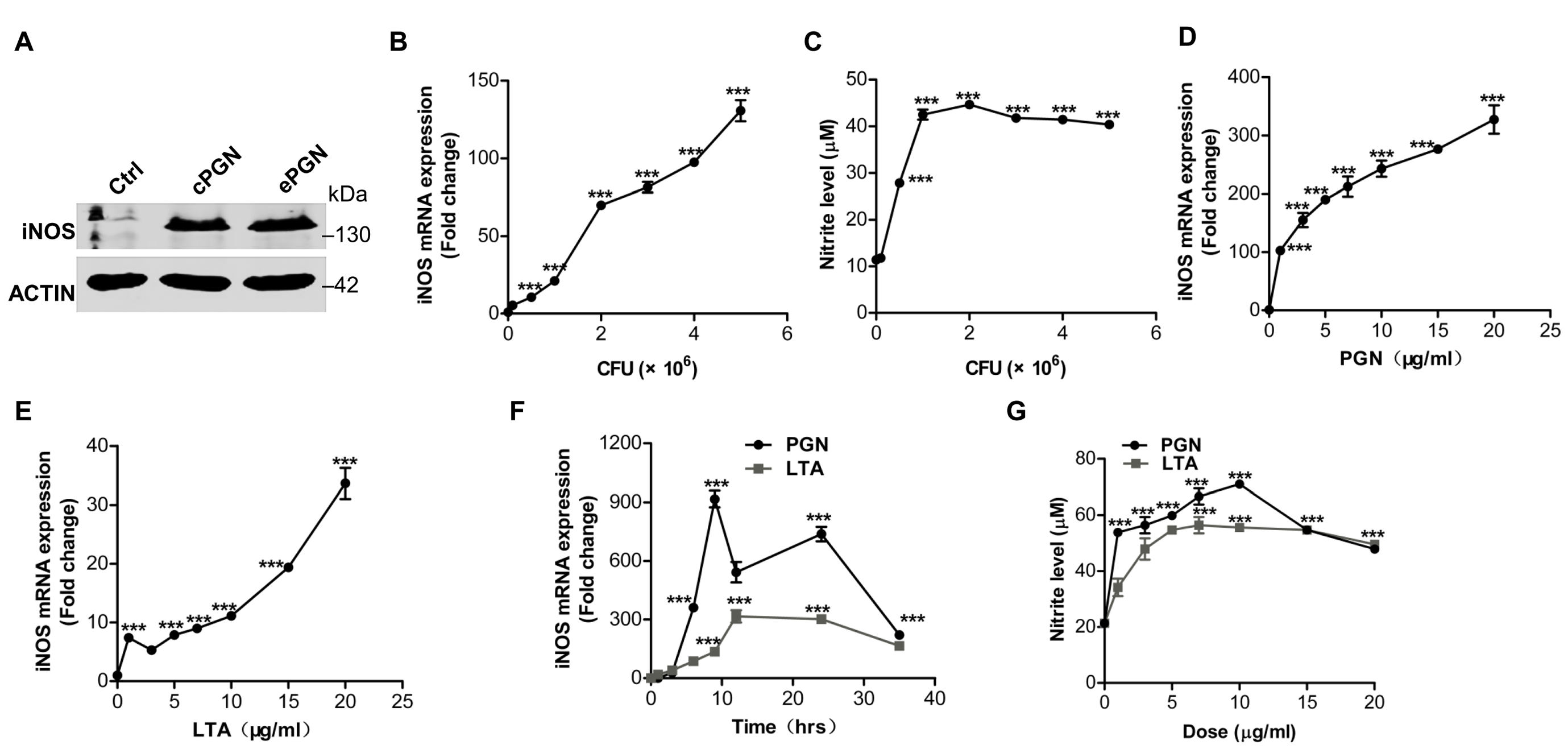

Supplement: Figure S3 — Staphylococcus aureus induces iNOS and NO release. (A) Western blot of iNOS in primary peritoneal macrophages stimulated by 10 µg/ml commercial PGN (cPGN) and PGN purified form S.aureus CMCC(B)26003 (cPGN) for 24 hours. (B) iNOS mRNA expression in RAW264.7 cells treated with different doses of heat-inactivated S.aureus for 24 hours. (C) NO production induced by different doses of heat-inactivated S.aureus for 24 hours in RAW264.7 cells. (D&E) Quantification of iNOS mRNA in RAW264.7 cells treated with different doses of PGN (D) or LTA (E) for 24 hours. (F) iNOS mRNA expression in RAW264.7 cells treated with 10 µg/ml PGN or LTA for various times. (G) NO production by different doses of PGN or LTA for 24 hours in RAW264.7 cells. ***P<0.001. P values were determined by one-way ANOVA. All data are means ± SEM of n = 3 and representative of two independent experiments. (TIF) [file ppat.1003918.s003.tif]

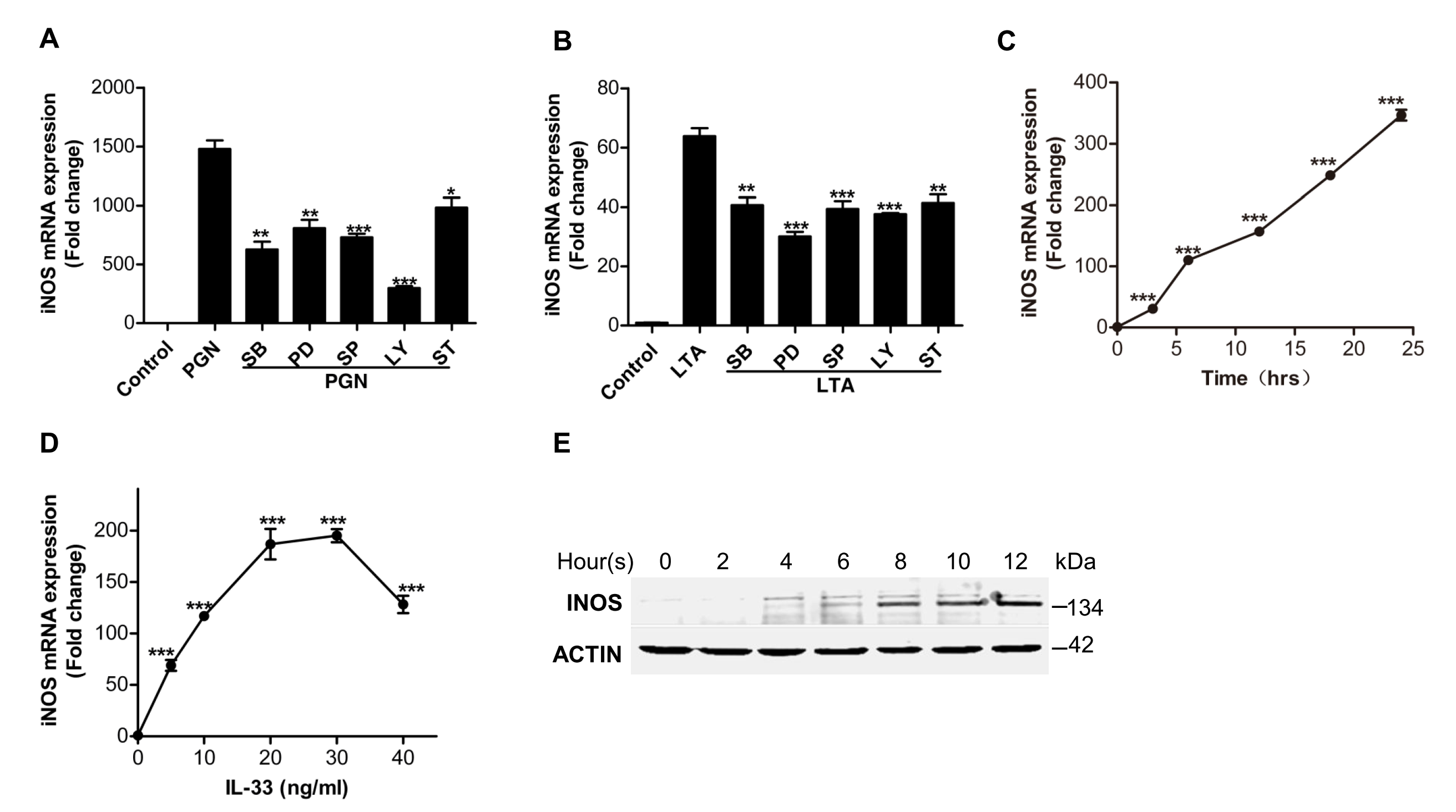

Supplement: Figure S4 — PGN, LTA and processed IL-33 induces iNOS expression in macrophages. (A&B) iNOS mRNA expression induced by 10 µg/ml PGN (A) or LTA (B) in the presence or absence of different inhibitors in RAW264.7 cells. SB: p38 MAPK inhibitor SB202190 (5 µM); PD: MEK1 inhibitor PD98059 (20 µM); SP: JNK inhibitor SP600125 (15 µM); Ly: AKT inhibitor Ly294002 (50 µM); ST: STAT3 inhibitor S3I-201 (50 µM). (C&D) iNOS mRNA expression induced by 30 ng/ml process IL-33 for different times (C) or by different dose of process IL-33 for 24 hours (D) in RAW264.7 cells. (E) Western blot of iNOS induced by 30 ng/ml process IL-33 for different times in RAW264.7 cells. * P<0.05, **P<0.01 and ***P<0.001. P values were determined by one-way ANOVA. All data are means ± SEM of n = 3 and representative of two independent experiments. (TIF) [file ppat.1003918.s004.tif]

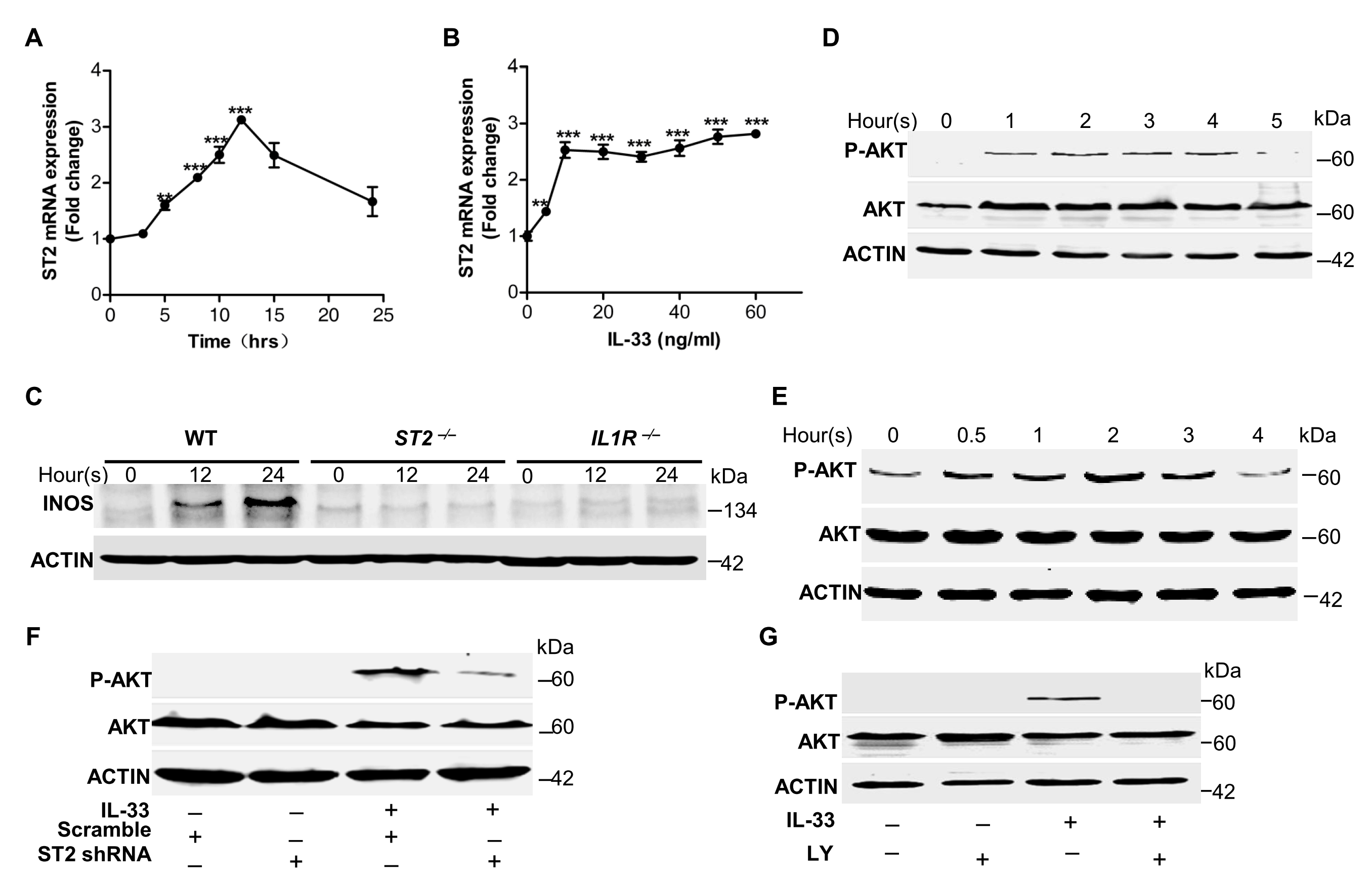

Supplement: Figure S5 — IL-33 induces iNOS via the activation of ST2-AKT-β-catenin in macrophages. (A&B) Quantification of ST2 mRNA expression in macrophages treated with 30 ng/ml IL-33 for different times (A) or different doses of IL-33 for 24 hours (B). (C) Western blot of iNOS in WT, ST2−/− and IL1R−/− BMDMs treated with 30 ng/ml processed IL-33(Ser109-Ile266) for different times. (D&E) Western blot of AKT phosphorylation induced by 30 ng/ml processed IL-33 for different times in RAW264.7 cells (D) or primary peritoneal macrophages (E). (F) AKT phosphorylation induced by 30 ng/ml processed IL-33 before and after ST2 was knocked down in RAW264.7 cells. (G) AKT phosphorylation induced by 30 ng/ml processed IL-33 in the presence or absence of AKT inhibitor Ly294002 (50 µM) in RAW264.7. **P<0.01 and ***P<0.001. P values were determined by one-way ANOVA. All data are means ± SEM of n = 3 and representative of two independent experiments. (TIF) [file ppat.1003918.s005.tif]

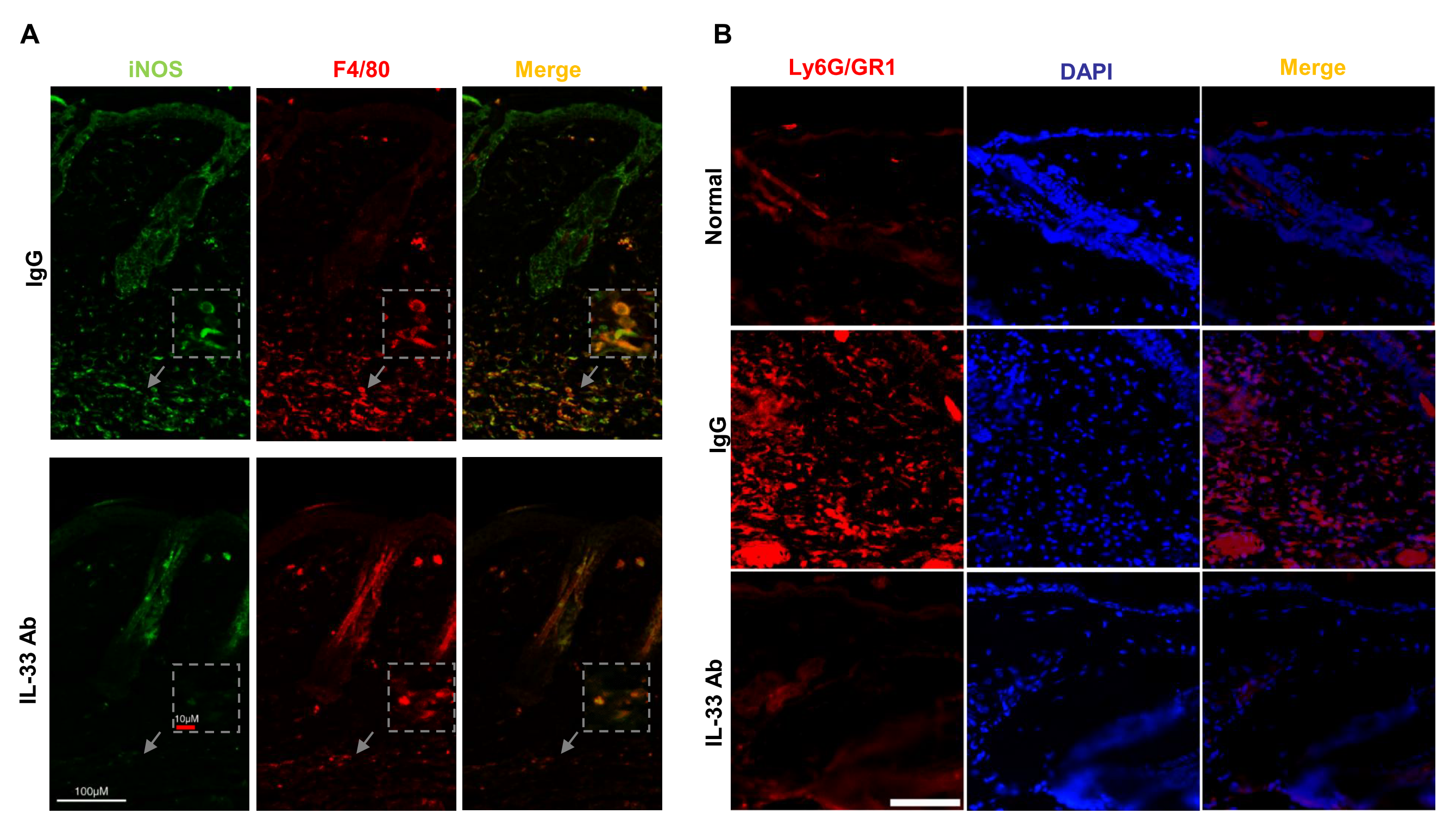

Supplement: Figure S6 — IL-33 neutralization decreases iNOS in macrophages and the recruitment of neutrophils in skin lesions. (A) Immunofluorescence analysis of iNOS and F4/80 (macrophage marker) in S.aureus-infected skin before and after IL-33 was neutralized. Red scale bar represents 10 µm and white scale bars represent 100 µm. The arrow designates region of 200× magnification shown in inset. (B) Immunofluorescence analysis of Ly6G/GR1 (neutrophil marker) in S.aureus-infected skin before and after IL-33 was neutralized. Scale bar represents 100 µm. (TIF) [file ppat.1003918.s006.tif]

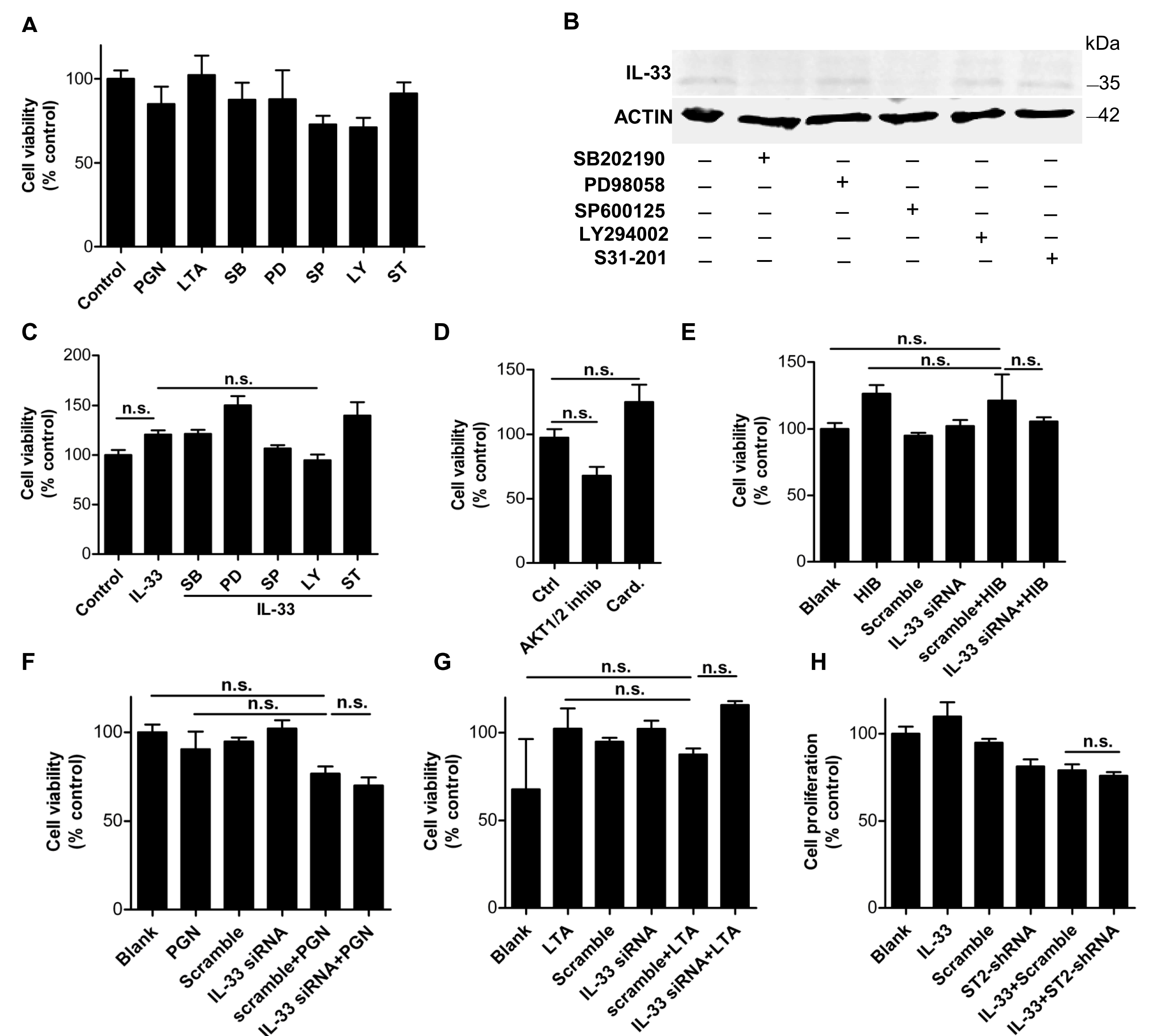

Supplement: Figure S7 — The cytotoxicity of inhibitors and shRNAs. (A) Cell viability of PGN, LTA or different inhibitors in macrophages RAW264.7 by MTT analysis. PGN: 10 µg/ml; LTA: 10 µg/ml; SB: p38 MAPK inhibitor SB202190 (5 µM); PD: MEK1 inhibitor PD98059 (20 µM); SP: JNK inhibitor SP600125 (15 µM); Ly: AKT inhibitor Ly294002 (50 µM); ST: STAT3 inhibitor S31-201 (50 µM). (B) Inhibitors failed to induce IL-33 protein in RAW264.7 cells by western blot analysis. (C&D) Cell viability of RAW264.7 cells treated with IL-33 in the presence or absence of different inhibitors. IL-33: 30 ng/ml; SB: p38 MAPK inhibitor SB202190 (5 µM); PD: MEK1 inhibitor PD98059 (20 µM); SP: JNK inhibitor SP600125 (15 µM); Ly: AKT inhibitor Ly294002 (50 µM); ST: STAT3 inhibitor S31-201 (50 µM); AKT1/2 specific inhibitor (8 µM); Card: β-catenin inhibitor cardamonin (9 µM). (E–H) Cell viability of RAW264.7 cells treated with 106 CFU heat-inactivated S.aureus (E) or PGN(F) or LTA (G) before and after IL-33 was knocked down or treated with IL-33 before and after ST2 was knocked down (H). n.s. no significance. P values were determined by one-way ANOVA. All data are means ± SEM of n = 3 and representative of two independent experiments. (TIF) [file ppat.1003918.s007.tif]
